# Bioactive compounds in coffee and their role in lowering the risk of major public health consequences: A review

**DOI:** 10.1002/fsn3.3848

**Published:** 2023-11-22

**Authors:** Markos Urugo Makiso, Yetenayet Bekele Tola, Onwuchekwa Ogah, Fitsum Liben Endale

**Affiliations:** ^1^ Department of Food Science and Postharvest Technology College of Agricultural Sciences Wachemo University Hossana Ethiopia; ^2^ Department of Postharvest Management College of Agriculture and Veterinary Medicine Jimma University Jimma Ethiopia; ^3^ Department of Applied Biology Ebonyi State University Isieke Nigeria; ^4^ Department of Public Health College of Medicine and Health Sciences Wachemo University Hossana Ethiopia

**Keywords:** caffeine, chlorogenic acid, coffee aroma, health benefits, trigonelline

## Abstract

This article addresses the bioactive components in coffee aroma, their metabolism, and the mechanism of action in lowering the risk of various potential health problems. The main bioactive components involved in the perceived aroma of coffee and its related health benefits are caffeine, chlorogenic acid (CGA), trigonelline, diterpenes, and melanoids. These compounds are involved in various physiological activities. Caffeine has been shown to have anticancer properties, as well as the ability to prevent the onset and progression of hepatocellular carcinoma and to be anti‐inflammatory. CGA exhibits antioxidant action and is implicated in gut health, neurodegenerative disease protection, type 2 diabetes, and cardiovascular disease prevention. Furthermore, together with diterpenes, CGA has been linked to anticancer activity. Trigonelline, on the other side, has been found to lower oxidative stress by increasing antioxidant enzyme activity and scavenging reactive oxygen species. It also prevents the formation of kidney stones. Diterpenes and melanoids possess anti‐inflammatory and antioxidant properties, respectively. Consuming three to four cups of filtered coffee per day, depending on an individual's physiological condition and health status, has been linked to a lower risk of several degenerative diseases. Despite their health benefits, excessive coffee intake above the recommended daily dosage, calcium and vitamin D deficiency, and unfiltered coffee consumption all increase the risk of potential health concerns. In conclusion, moderate coffee consumption lowers the risk of different noncommunicable diseases.

## Graphical abstract

Coffee aroma and health benefits. Health consequences of bioactive compounds in coffee. Coffee and health.

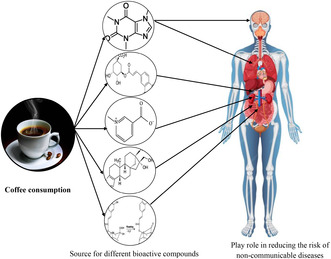

## 1 INTRODUCTION

Coffee is the most popular beverage consumed worldwide and has several positive health and economic effects (Esteban et al., 2020; Haile et al., 2020; Wu et al., 2022). It is consumed daily by millions of individuals who enjoy its stimulating and refreshing qualities as well as its pleasant flavor and aroma (Farag et al., 2022; Pohl et al., 2012). Over 166 million bags of coffee were consumed in the fiscal year 2020–2021 globally, with an estimated daily consumption of over 1.6 billion cups (Farag et al., 2022; Statista, 2022). Global demand for coffee climbed steadily by 2.2% on average between 2016–2017 and 2019–2020 (Mas Aparisi, 2021). Several variables, including the improvement in bean quality, the growth of specialty coffee shops, and the spread of literature relating coffee use to health advantages, could explain the ongoing rise in coffee demand (Celli & Camargo, 2019).

Numerous scientific studies have shown that drinking coffee lowers the risk of certain health complications, including type II diabetes, heart disease, liver cirrhosis, obesity, and cancer (Iriondo‐dehond et al., 2020; Kusumah & Mejia, 2022; Núñez et al., 2020; Wu et al., 2022). Coffee's high antioxidant activity and bioactive components are typically responsible for the beverage's positive health effects (Esteban et al., 2020; Haile et al., 2020; Iriondo‐dehond et al., 2020; Núñez et al., 2020; Saud & Salamatullah, 2021). Coffee, both green and roasted, contains a variety of bioactive substances, including phenolic acids, primarily chlorogenic acids (CGAs), lactones (CQAs, caffeoylquinic acids, FQAs, feruloylquinic acids, and diCQAs, dicaffeoylquinic acids with at least three isomers per class), methylxanthines (caffeine, theophylline, and theobromine), diterpenes (cafestol and kahweol; Haile et al., 2020; Jeszka‐skowron et al., 2020; Saud & Salamatullah, 2021; Wu et al., 2022). Some of these elements play crucial roles as precursors to the coffee aroma both before and after roasting (Bastian et al., 2021; Haile et al., 2020).

The primary ingredients for the aroma and bioactivity of coffee include caffeine (CAF), CGA, trigonelline (TGA), nicotinic acid, and sucrose (Jeszka‐skowron et al., 2020; Stefanello et al., 2018). Coffee aroma is one of the most distinguishing qualities of this commodity and the most important factor influencing consumers' acceptance and preference (Caporaso et al., 2018; Turan et al., 2021). The chemical makeup of the bean, genetic strain, geographic location, climate, annual rainfall, agricultural practice, and the processing method used are all directly related to the aroma of coffee (Procida et al., 2020). Species differences in the coffee may bring difference in the chemical makeup of the coffee. There have been more than 120 coffee species discovered so far, but only two of them, *Coffea arabica* (Arabica) and *Coffea canephora* (Robusta), supply 99% of the world's coffee needs (Angeloni et al., 2021; Farag et al., 2022; Moeenfard & Alves, 2020; Zhang et al., 2020).

Arabica and Robusta beans have different chemical makeup and consequently they have different flavor profiles. Arabica beans produce a superior taste in the cup, more flavorful and complex than Robusta beans. On the other hand, Robusta often produce a more bitter beverage with a musty flavor and more body (Ogutu et al., 2022; Seninde & Chambers IV, 2020; Wongsa et al., 2019). The differences in sensory qualities may influence consumers' preferences, feelings, or attitudes regarding coffee consumption (Seninde & Chambers IV, 2020). Nowadays consumers are increasingly interested in their diet and health. However, it remains unclear what consumers think about coffee's health advantages. Only 16% of Americans who drink coffee are aware of its health advantages, and 66% choose to limit their caffeine intake. Many European customers are perplexed about how coffee affects their health, with 49% of them believing that it does (Samoggia & Riedel, 2019). As a result, this article aimed to review recent scientific reports on aroma impact bioactive compounds of Arabica and Robusta coffees and their health benefits.

## 2 METHODOLOGY

**FIGURE 1:**
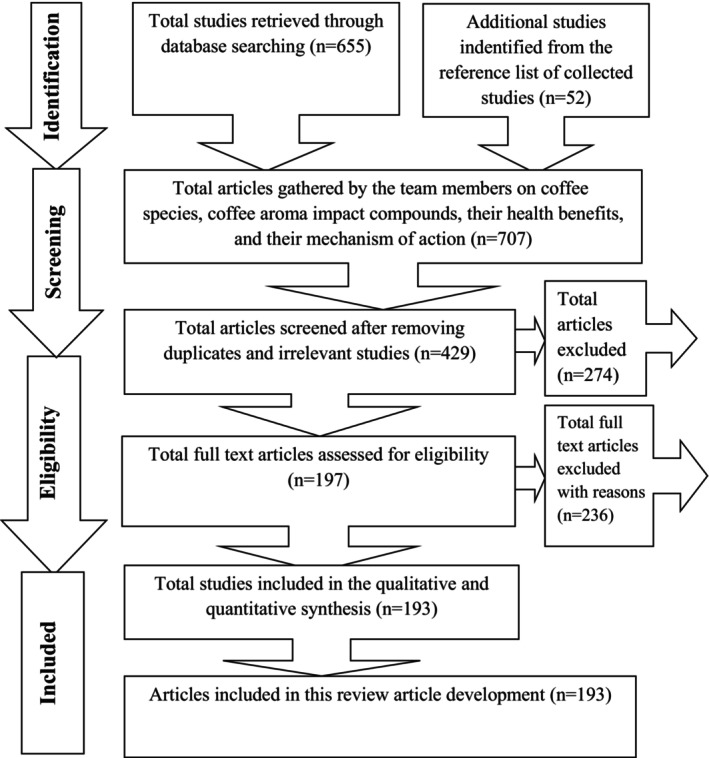
A schematic diagram detailing the article search and selection.

The most relevant articles on the coffee aroma impact compounds and their health benefits were explored from related databases such as Google Scholar, Web of Science, PubMed, Science Direct, and Virtual Library of Health. Key terms used in the data search were “coffee”, “coffee species”, “health benefits of coffee”, “aroma active compounds of coffee”, “aroma active compounds of Arabica and Robusta coffee”, “health benefits of caffeine, CGA, trigonelline, diterpenes, and melanoids”. Furthermore, the references of selected publications were examined to obtain further relevant articles. As shown in Figure 1, the search criteria included all peer‐reviewed articles published in English between 2004 and September 2023. A total of 707 articles were collected. Articles that passed the title and abstract screening process were then read in their entirety by two members to determine their eligibility to be included in the review. During the eligibility screening, articles that met the following criteria were included in the review: (1) has been peer reviewed; (2) has been published in English; (3) has been published between 2004 and 2023; and (4) the full text of the article is available. In the screening step, 510 articles that failed to meet the eligibility criteria were rejected, and the remaining 197 articles were identified as eligible and used as information sources for this review article.

## 3 COFFEE SPECIES

The coffee plant is a woody perennial evergreen shrub with white flowering plants that produce fruits called “cherries” that contain two seeds known as “coffee beans” (Bliss, 2017). Economically important coffee species originate from Africa and belong to the family Rubiaceae, genus *Coffea* (Adronikos, 2019). *Coffea arabica* and *C. canephora* are the two most traded and consumed coffee species globally. *Coffea arabica* accounts for 70% of the world's coffee production, and *C. canephora* and *Coffea liberica* cover the remaining share (Angeloni et al., 2021; Nunez et al., 2020, 2021). Coffee grows in over 70 countries in tropical regions (Hunt et al., 2020). Specifically, the tropics of Cancer and Capricorn, known as the “bean belt”, such as Ethiopia, Brazil, Costa Rica, and Indonesia, are preferable for coffee growth (Bliss, 2017). It takes 3 years to develop from seed germination to first flowering and fruit production (Rodrigues et al., 2013). The growth requirement varies for different coffee species but generally, they require a well‐distributed annual rainfall, with temperature between 15°C and 30°C and dry season for up to 5 months. *Coffea arabica* and *C. canephora* are coffee species adapted to tropical highlands and lowlands, respectively (Angeloni et al., 2021; Pohlan & Janssens, 2010).

## 4 COFFEE AROMA IMPACT COMPOUNDS

The pleasurable flavor and aroma of coffee are largely attributed to a variety of biochemical components. Both genetic and environmental factors have an impact on these biochemical components (Getachew et al., 2020). Green coffee contains different components, namely, methylxanthines (caffeine, theophylline, and theobromine), CGA, trigonelline, lipids, carbohydrates, especially insoluble polysaccharides such as cellulose, hemicelluloses, sugars including arabinose, fructose, galactose, glucose, raffinose, sucrose, stachyose, free amino acids, fiber, water, and minerals (Mengistu et al., 2020; Pimpley et al., 2020). In addition, they are known to have nonvolatile fractions of aliphatic acids such as citric acid, malic acid, quinic acid, and acetic acid along with the volatile ones such as acetic acid, propanoic acid, butanoic acid, hexanoic acid, and decanoic acid (Pimpley et al., 2020). Caffeine, CGA, and sucrose are the major components and precursors of coffee cup quality (Tolessa et al., 2019). Generally, nonvolatile components of coffee are responsible for taste sensations of the coffee like sourness, bitterness, and astringency, whereas volatile compounds of coffee are responsible for the aroma of the coffee (Buffo & Cardelli‐freire, 2004). Due to their differing chemical makeups, roasted Arabica and Robusta green beans have different flavor characteristics (Seninde & Chambers IV, 2020).

### 4.1 Nonvolatile components of coffee

Nonvolatile coffee chemicals include, for instance, lipids, CGAs, organic acids, some minerals, melanoids, and nitrogen‐containing substances like trigonelline and caffeine (Cordoba et al., 2020). Caramelization, Maillard reaction, and pyrolysis occur during high‐temperature roasting. They degrade some nonvolatile components like proteins and carbohydrates and give rise to the formation of various bioactive compounds with high and medium volatility that impart the aroma and flavor of coffee (Farah, 2012).

#### 4.1.1 Caffeine (1,3,7‐trimethylxanthine; C_8_H_10_N_4_O_2_ )

**FIGURE 2:**
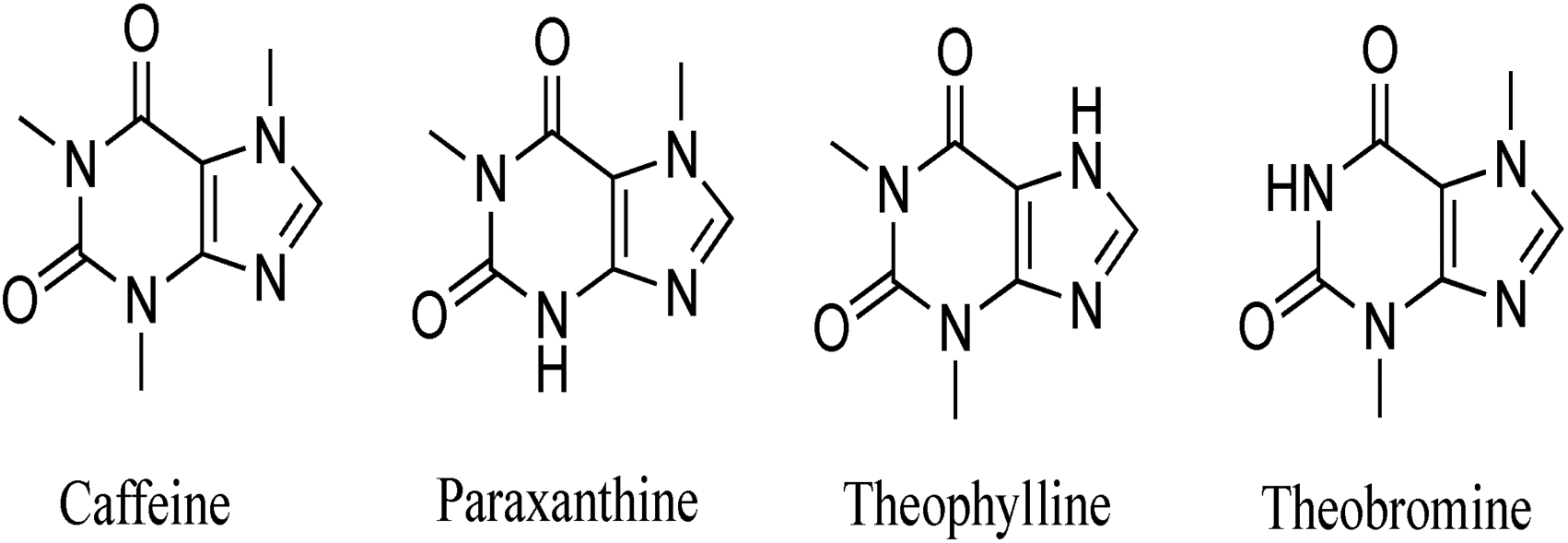
Chemical structure of caffeine and its metabolites (Anastasiadi et al., 2021).

Caffeine is one of the pharmacologically active methylxanthines found in coffee. It has an effect on the central nervous system, heart, gastrointestinal system, and respiratory systems (DePaula & Farah, 2019; Jeszka‐skowron et al., 2020). Caffeine is the major compound in coffee responsible for psychostimulating sensations and imparts body, strength, and 10% of the perceived bitterness of brewed coffee (Sharma, 2020; Sunarharum, 2016). However, it is colorless and odorless at room temperature and dissolves in boiled water (DePaula & Farah, 2019). It is found in twice the amount higher in *C. canephora* (1.5%–4.0%) than in *C. arabica* (0.7%–1.6%; Jeszka‐skowron et al., 2020). Caffeine is relatively heat stable and not affected by roasting temperature, but slight loss occurs as a result of sublimation (Farah, 2012). Brewed coffee contains 135 mg of caffeine per cup (237 mL, 8 oz); instant coffee contains 76–106 mg; and decaffeinated instant contains 5 mg of caffeine per cup (Morgan et al., 2013). However, according to the study, commercial espresso coffees have a 6‐fold range of caffeine levels, a 17‐fold range of CQA contents, and a fourfold range of CQA–caffeine ratio. These variations are due to batch‐to‐batch differences in bean composition, possible Arabica–Robusta bean blending, and roasting and grinding procedures (Crozier et al., 2012). These differences significantly limit the validity of epidemiological study conclusions. The chemical structure of caffeine and its metabolites are indicated below in Figure 2.

#### 4.1.2 Chlorogenic acid (5‐CQA; C_16_H_18_O_9_ )

**FIGURE 3:**
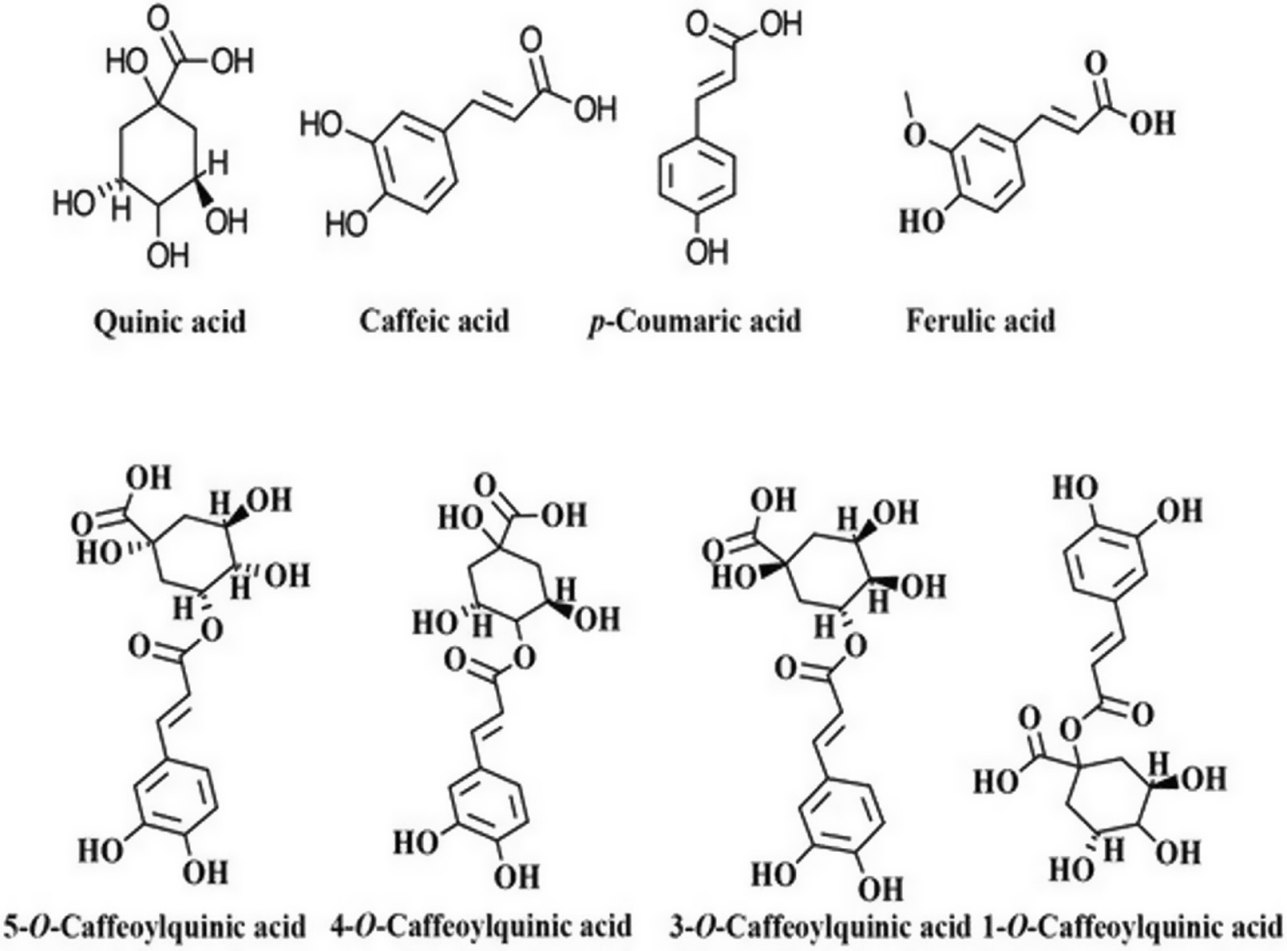
Chemical structure of chlorogenic acids and its derivatives (Hernandez‐Estrada, 1985).

Chlorogenic acid is a derivative of trans‐cinnamic acids (caffeic, ferulic, or p‐coumaric) and quinic acid by esterification reaction (Pinheiro et al., 2020). It is the major polyphenol of coffee and comprises 12%–18% of the dry matter basis of green coffee. The compound has antioxidant properties and also has both pharmacological and physiological effects (Farah & Lima, 2019; Pinheiro et al., 2020). The chemical structure of CGA and its derivatives are indicated below in Figure 3. There are three different classes of CGAs that cover 80% of the total CGA content of coffee; these are CQA, diCQA, and FQA (Pimpley et al., 2020). During roasting, CGAs degrade into lower molar compounds called lactones and phenolic derivatives like caffeic acids. These compounds further hydrolyze or isomerize to impart aroma and taste such as bitterness and astringency to the coffee (Pinheiro et al., 2020). Depending on the type of coffee and brewing method, one cup (200 mL) of coffee contains between 20 and 675 mg of CGAs (Jeszka‐Skowron et al., 2016).

#### 4.1.3 Trigonelline (*N*‐methyl nicotinic acid; C_7_H_7_NO_2_ )

**FIGURE 4:**
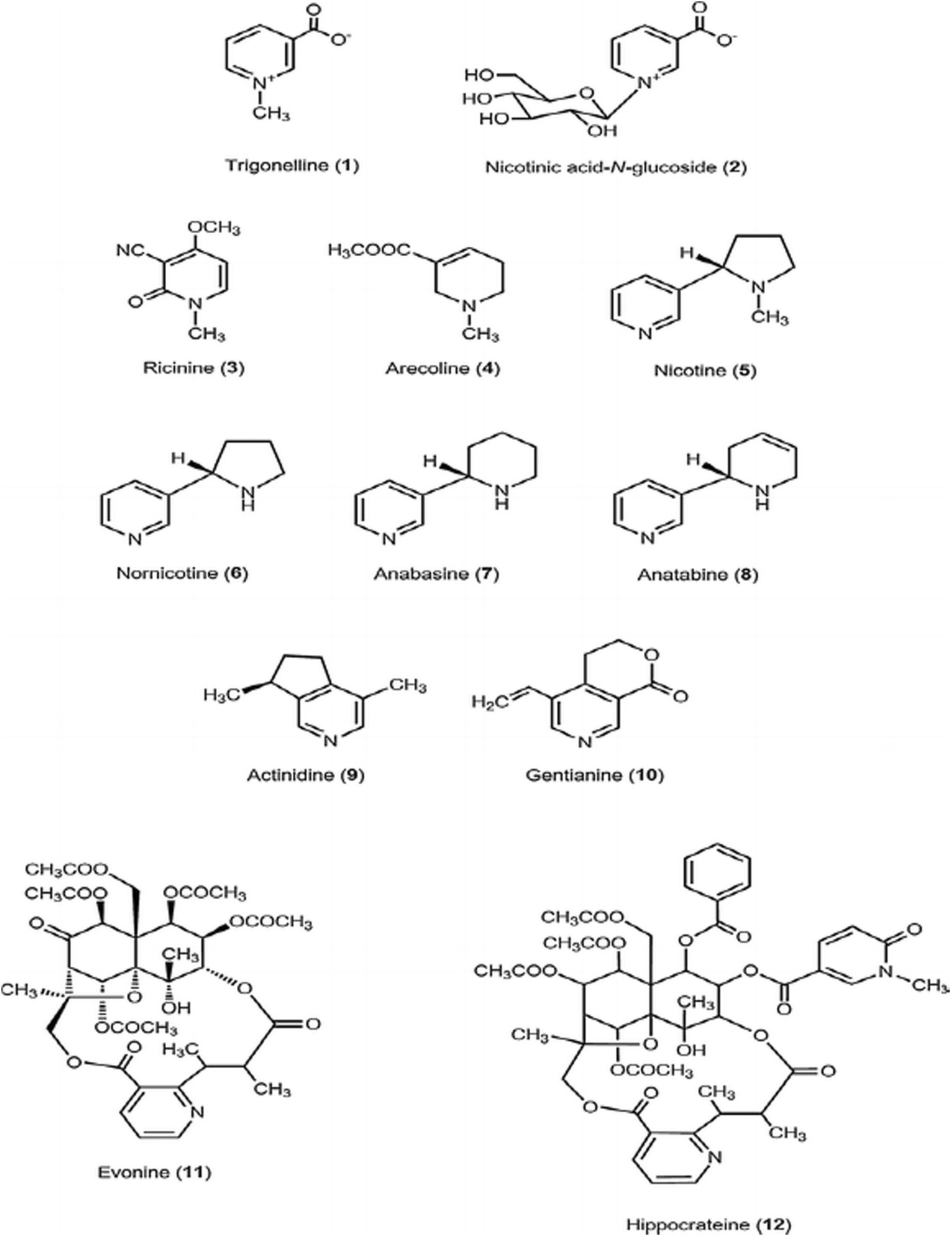
Structure of trigonelline and related nicotinic acid metabolites (Ashihara et al., 2015).

It is a plant alkaloid formed by methylation of the nitrogen atom of nicotinic acid by the catalytic effect of NaMN nucleotidase, NaR nucleotidase, and nicotinate methyl transferase (Arai et al., 2015; Mohamadi et al., 2017). It is found in the range of 0.6%–1.3% in Arabica coffee and 0.3–0.9% in Robusta green bean (Mohamadi et al., 2017). Trigonelline has pharmacological effect but its level significantly decreases after roasting (Mengistu et al., 2020). During coffee roasting, trigonelline induces demethylation of niacin (Vitamin B3 or nicotinic acid), volatile coffee constituents such as pyridines, pyrrole derivatives, methyl esters of nicotinic acid, and other compounds responsible for coffee aroma (Jeszka‐skowron et al., 2020; Mohamadi et al., 2017). Figure 4 below shows the chemical structure of trigonelline and related nicotinic acid metabolites. Table 1 presents the nonvolatile aroma impact biochemical compositions of Arabica and Robusta coffee and their role in the perceived sensation.

**TABLE 1:** Aroma impact compounds of roasted Arabica and Robusta coffees and their role in the perceived sensation.

Nonvolatile compounds	Their amount		Role on perceived sensation
	*Coffea arabica*	*Coffea canephora*	
Caffeine	0.6%–1.2% dwb*	2.2%–2.8% dwb	Responsible for bitterness, body, and strength of brewed coffee
Melanoids/humic acid	16.0%–17.0% dwb	16.0%–17.0% dwb	Important for roasted coffee color
Carboxylic acids			
Citric acid			Essential for sourness of coffee
Malic acid			
Acetic acid			
Chlorogenic acids			
Cinnamic acid	3.4%–7.24% dwb	5.17%–14.4% dwb	All are responsible for astringency
Caffeic acid			
Ferulic acid			
Isferulic acid			
Sinapic acid and their degradation product Quinic acid			
Trigonelline			
Contains two derivatives namely: nicotinic acid and *N*‐methylnicotinamide	0.6%–1.3% dwb	0.3%–0.9% dwb	Have role on bitterness of coffee and nicotinic acid has nutritional importance
Carbohydrates			
Arabinogalactose	43.0%–45.0% dwb	46.9%–48.3% dwb	Contribute to the retention of volatiles and play role in viscosity of coffee brew
Cellulose			
Hemicelluloses			
Pectins			
Lipids			
Triglycerides	14.5%–20.0% dwb	11.0%–16.0% dwb	Important for viscosity of brew, foam stability, flavor retention
Terpenes			
Tocopherols			
Sterols			
Minerals			
Potassium around 40% W/W manganese, iron, copper	3.5%–4.5% dwb	4.6%–5.0% dwb	They catalyze different reactions during coffee storage and roasting

*Source*: Campa et al. (2004), Sanlier et al. (2019); Yeretzian et al. (2019).

Abbreviation: dwb*, dry weight basis.

#### 4.1.4 Lipids

**FIGURE 5:**
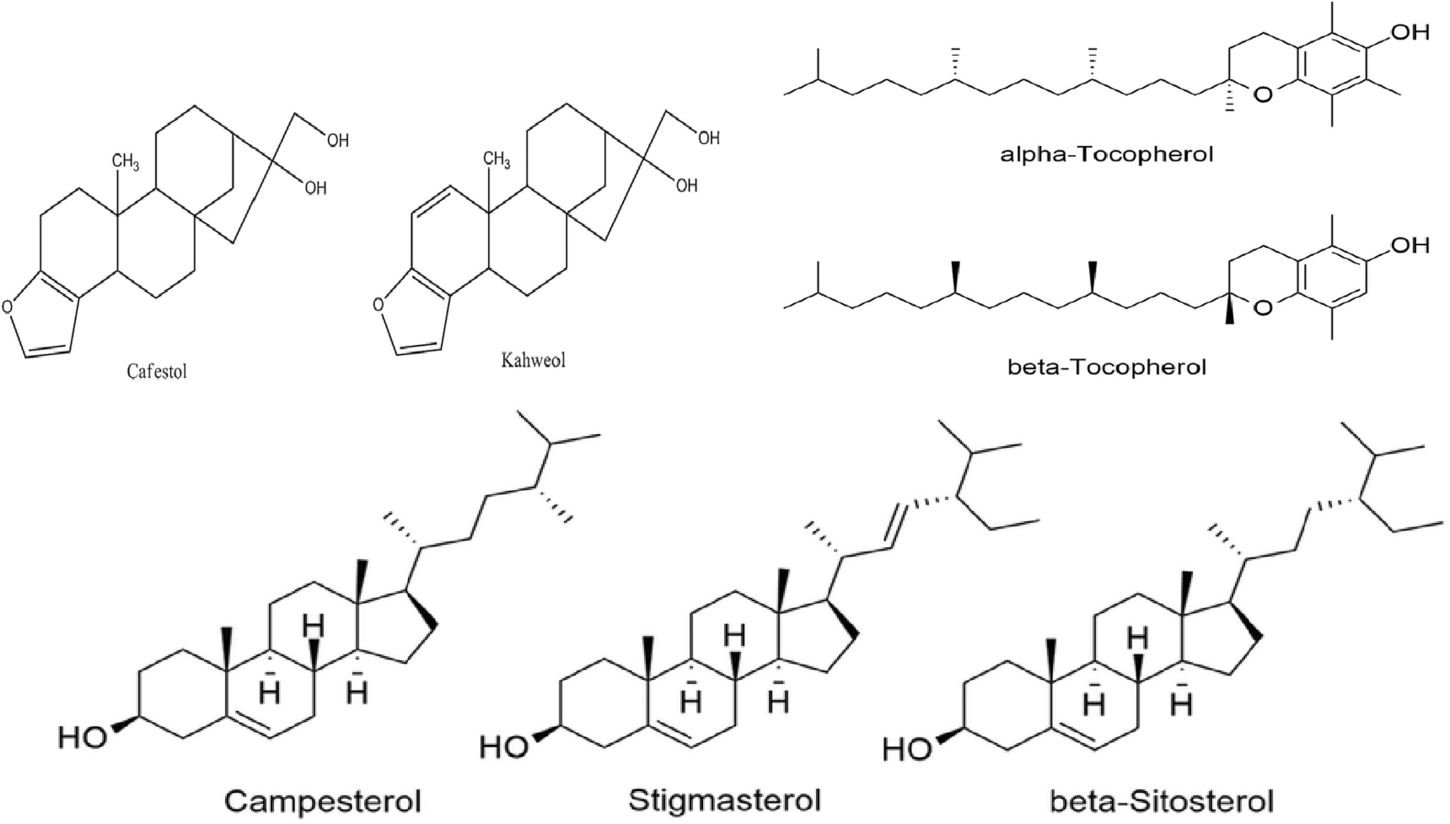
Chemical structure of common lipids in coffee (Silva et al., 2012; Vandeponseele et al., 2020).

Lipids affect the stability of the foam in the drink and are important for flavor retention. In addition to having a taste and smell, lipids are known to retain volatile chemicals within the foam layer. Growing circumstances have an impact on lipids, and studies have shown that these variables have a negative link with lipid accumulation in coffee beans (Godos et al., 2014; Ren et al., 2019). Arabica coffee has a lipid content that is substantially higher than Robusta coffee at 8%–18% of its dry matter, with triglycerides making up 75% of this content and esters of fatty acids diterpenes making up 20% (Kitzberger et al., 2016). The main diterpenes in coffee oil include cafestol, kahweol, and 16‐O‐methyl cafestol (16‐OMC), and a cup of unfiltered coffee has 3–6 mg of each of these diterpenes (Ren et al., 2019). Kahweol, cafestol, and 16‐OMC levels varied among various species of coffee, and this has made them a crucial tool for identifying them. Studies revealed that Arabica coffees grown under typical soil and climate circumstances and Arabica coffees with *C. canephora* introgressions have different diterpene contents (Kitzberger et al., 2013, 2016). The chemical structure of common lipids in coffee is presented in Figure 5.

#### 4.1.5 Melanoids

The Maillard reaction, sometimes referred to as the nonenzymatic browning reaction, is frequently experienced during the roasting of coffee and many other culinary items. When the temperature of roasting coffee beans reaches roughly 154°C, a complicated reaction known as the Maillard reaction occurs between amino compounds and reducing sugars (Golon et al., 2014; Hu et al., 2019). This reaction can also occur between aldehydes, ketones, peptides, proteins, and even ammonia. The polymers formed by this reaction are still a mystery; all that is now known about them is the chemical mechanism that results in low‐ and medium‐molecular‐weight products. Early stage, middle stage, and ultimate stage are three divisions that can be made of them. Hodge developed the Maillard reaction method in the 1950s, which was extensively used to describe how low‐molecular‐weight (LMW) Maillard reaction products are produced. It postulated the Maillard reaction's early phases (Golon et al., 2014; Hu et al., 2019; Iriondo‐dehond et al., 2021).

**FIGURE 6:**
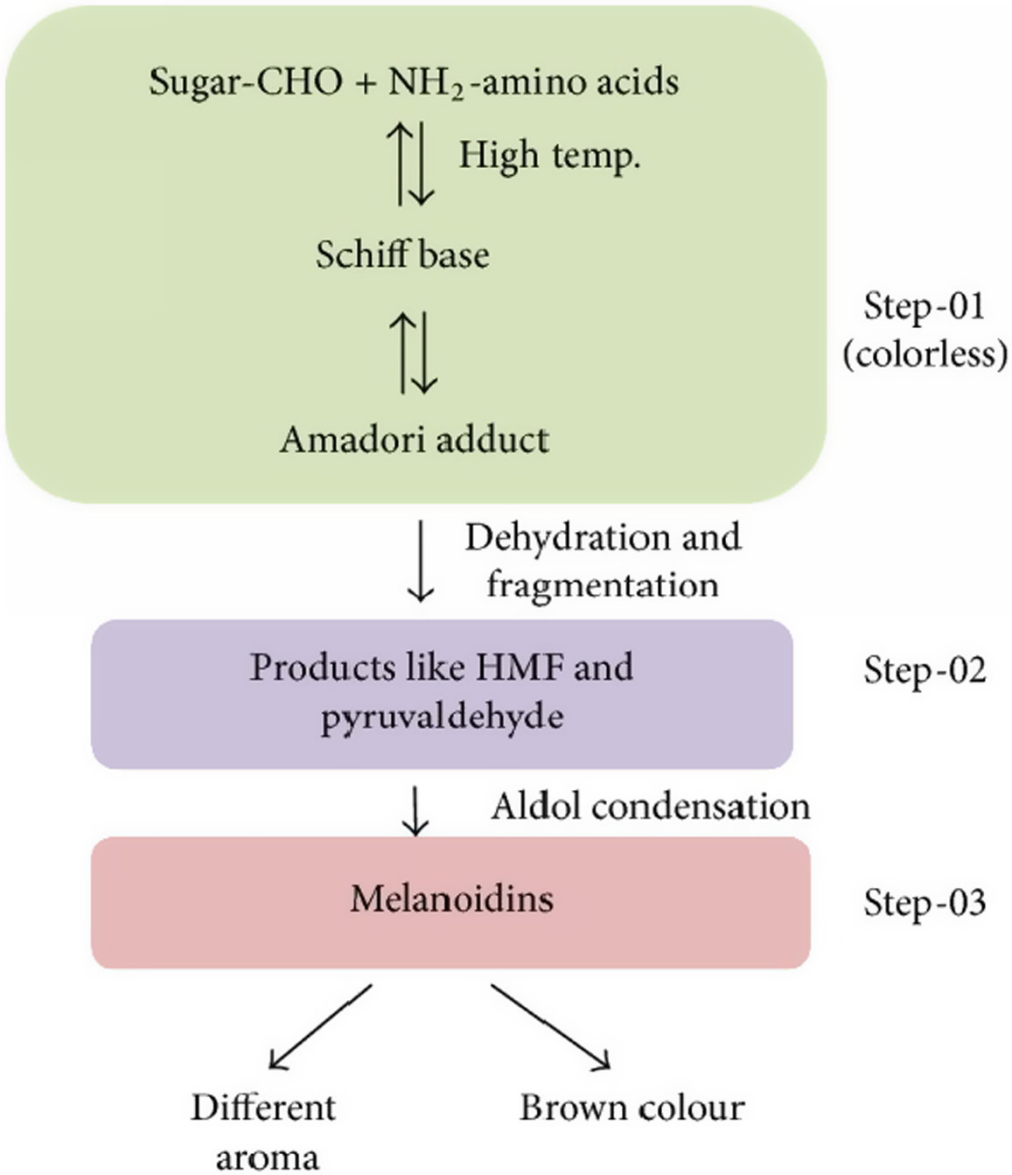
Schematic representation of melanoid formation due to Maillard reaction (Tamanna & Mahmood, 2015).

Early in the process, the amino group is combined with the carbonyl group of the reducing sugar, which is then dehydrated to create a Schiff base. The Schiff base is then cycled to create *N*‐glucosamine, which is then transformed into 1‐amino‐1‐deoxy‐2‐ketosaccharide through an Amadori rearrangement. Amadori compounds are converted to reducing ketones or furfural compounds by a series of reactions that also generate a large number of reactive intermediates (Nakamura & Kawaharada, 2021). Additionally, Strecker degradation may be applied to the condensate of the dicarbonyl compound and the amino acid created by the breakdown of the Amadori compound. Coffee's Maillard reaction can result in a variety of substances that are good for our health, particularly high molecular weight molecules (HMWM), also known as melanoidins (Bikaki et al., 2021; Hu et al., 2019; Iriondo‐dehond et al., 2021). Figure 6 depicts the schematic representation of the Maillard reaction steps in the formation of melanoids.

**FIGURE 7:**
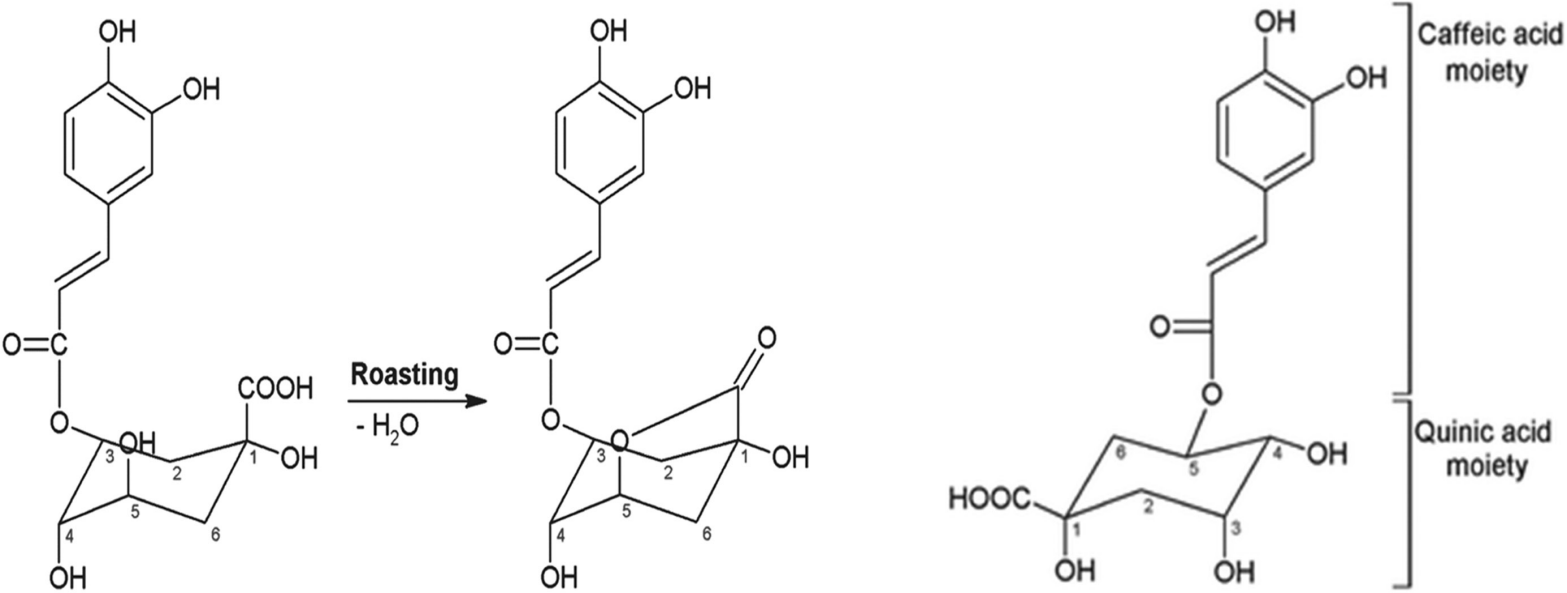
Chemical structure of melanoids (Moreira et al., 2012).

In addition to LMW chemicals, proteins, and polysaccharides, phenols, which take the form of glycosidic bonds, play a significant role in the synthesis of melanoidins and make up approximately 10% of the total weight (Lima, 2010). A study revealed that the primary mechanism for the addition of phenolic compounds to HMWM is transglycosylation reactions (Moreira et al., 2017). The most prevalent polysaccharides in coffee beans are galactomannan and type II arabinogalactan, which when baked with LMW, proteins, and phenolic chemicals produce melanoidins (Hu et al., 2019). An average coffee drinker (four cups/day) can acquire 1.5 g of melanoidins from this source. The estimated quantity of melanoidins per serving size for various coffee brew preparations ranges from 99 to 433 mg (Iriondo‐dehond et al., 2021). The chemical structure of melanoids is depicted in Figure 7 below.

### 4.2 Volatile aroma compounds of coffee

The aroma of coffee is affected by environmental factors such as climatic factors and soil factors, genetic predispositions of the coffee that contain a specific set of chemical precursors and bases for its aroma compounds, harvesting and postharvesting practices, sorting, storage, and transport. These parameters adjust the genetic predisposition and affect the biochemical composition of the green bean as it goes to roasting. Finally, the roasting conditions, grinding, and extraction process cumulatively alter the aroma of the coffee (Girmay et al., 2019; Yeretzian et al., 2019). During the roasting of coffee, different flavor and aroma compounds are formed due to Maillard, thermal degradation, and pyrolysis reactions. The Maillard reaction results in the formation of pyrroles, thiols, pyrazines, pyridines, thiophenes, and furans. Thermal degradation produces various products of caramelization and a diverse group of phenolic compounds. The pyrolysis reaction produces a diverse range of organic compounds, including alcohols, aldehydes, carboxylic acids, furans, ketones, pyrazines, and pyrroles (Toledo et al., 2016; Yeretzian et al., 2019).

One of the most crucial elements for determining coffee quality is the volatile portion of roasted coffee, and it might be significant at the time of purchase (Procida et al., 2020). As of now, approximately 1000 volatile compounds have been identified as part of the volatile composition of roasted coffee (Toledo et al., 2016; Wang, Pan, et al., 2022; Wang, Wang, et al., 2022; Yeretzian et al., 2019). The volatile fraction of the roasted arabica samples seems to be distinguished by the presence of high levels of pyridine, diacetyl, propyl formate, acetone, and 2,3‐pentanedione, whereas the Robusta samples appear to be distinguished by the presence of high levels of methylbutyrate, 2,3‐dimethylpyrazine, and 3‐hexanone (Procida et al., 2020). Table 2 below indicates a list of key volatile compounds of roasted Arabica coffee with their respective aroma descriptor.

**TABLE 2:** Volatile aroma compounds of roasted coffee.

Key odorants	Volatile compounds	Aroma descriptor
Aldehyde	2‐methylbutanal	Rancid, almond‐like, toasty
	2‐methylpropanal	Toasty, Caprylic, cheesy, dark chocolate, ethereal, fruity, malty, pungent
	3‐methylbutanal	Fruity, almond‐like, toasty, ethereal, chocolaty, peachy. fatty
	(E)‐2‐nonenal	Fatty, green, cucumber, citrus
	Acetaldehyde	Pungent, ethereal, fresh, lifting, penetrating, fruity, musty
	4‐methoxybenzaldehyde	Sweet, powdery, vanilla, anise, woody, coumarin, creamy
	Phenyl acetaldehyde	Sweet, fruity, honey, floral, fermented
	Propanal	Ethereal, pungent, earthy, alcoholic
Acid	2‐methylbutryic acid	Acidic, fruity, dirty, cheese
	3‐nethylbutryic acid	Cheesy, dairy, acidic, sour, pungent, fruity, stinky
Esters	Ethyl‐2‐methybutyrate	Fruit, berry
	Ethyl‐3‐methybutyrate	Fruity
Furan	Furfural (2‐((methylthio)methyl)furan; 2‐furfuryl methyl sulfide)	Sweet, brown, woody, bread, caramellic
	2‐furan, ethanol acetate/furfuryl acetate	Smoke, roast, onion, garlic, sulfurous, pungent, vegetable, horseradish
	2‐methylfuran	Onion, garlic, sulfurous, pungent, vegetable, creamy
	5‐methyl‐2‐furan carboxaldehyde/2‐methyl furfural	Burnt, ethereal (mild), gasoline, acetone, chocolate
	Furfuryl formate	Sweet, caramellic, bready, brown, coffee‐like
	Furfuryl methyl ether	Ethereal
	Furfuryl disulfide	Roasted coffee
Sulfur containing compounds	Dimethyl trisulfide bis(2‐methyl‐3‐furyl)disulfide	Sulfurous, coffee, roasted chicken, meaty, onion, cabbage
	Methanol	Boiled potato‐like, musty, tomato, earthy, vegetable, creamy
Thiols	3‐mercapto‐3‐methyl butyl formate	Green blackcurrant, herbal, fruity, roasted, sweaty
	2‐furfuryl thiol	Roasted (coffee‐like), sulfurous
	2‐methyl‐3‐furanthiol	Sulfurous, meaty, fishy, metallic, boiled
	3‐mercapto‐3‐methyl butyl acetate	Roasty, fruity, sulfurous, sweet
	3‐methyl‐2‐butane‐1‐thiol	Sulfurous, smoky, leek, onion
	Methanethiol	Rotten eggs, meat or fish, cabbage, garlic, cheesy
Thiophene	3‐methylthiophene	Fatty, wine
Thiazole	2,4‐dimethyl‐5‐ethylthiazole	Nutty, roasted, meaty, earthy
Furanone	Dihydro‐2‐methyl‐3(2H)‐furanone	Sweet, bread, buttery, natty
	2‐ethyl‐4‐hydroxy‐5methyl‐3(2H)‐furanone (Homofuraneol)	Sweet, caramel, candy
	3‐hydroxy‐4,5‐dimethyl‐2(5H)‐furanone (Sotolone)	Extremely sweet, strong caramel, maple, burnt sugar, coffee
	4‐hydroxy‐2,5‐dimethyl, 3(2H)‐furanone (furaneol)	Sweet, candy, caramel, strawberry, sugar
	5‐ethyl‐3‐hydroxy‐4‐methyl‐2(5H)‐furanone (abhexon)	Seasoning‐like, caramel‐like
	5‐ethyl‐4‐hydroxy‐2‐methyl‐3 (2H)‐furanone	Sweet, caramel, bread, maple, brown sugar, burnt
Ketone	1‐octen‐3‐one	Herbal, mushroom, earthy, musty, dirty
	2,3‐hexadione	Burnt, buttery, caramel, chocolate cream, creamy, fruity, oily, pear, sweet
	2,3‐butanedione	Buttery, creamy, fatty, oily, sweet, vanilla
	2,3‐pentandione	Butter, caramel, creamy, penetrating, sweet
	4‐(4‐hydroxyphenyl)‐2‐butanone	Sweet fruity, berry, jam, raspberry, ripe, floral (raspberry ketone)
	1‐(2‐furanyl)‐2‐butanone	Rummy
Norisoprenoid	(E)‐β‐damascenone	Honey‐like, fruity, apple, rose, honey, tobacco, sweet
Phenolic compounds	Guaiacol	Phenolic, burnt, smoke, spice, vanilla, woody
	4‐ethyl guaiacol	Spicy, smoky, bacon, phenolic, clove
	4‐vinyl guaiacol	Spicy, dry woody, fresh amber, cedar, roasted peanut
	Vanillin	Sweet, vanilla, creamy
Pyrazines	2,3‐dimethylpyrazine	Nutty, coffee, peanut butter, walnut, caramel, leather
	2,5‐dimethylpyrazine	Cocoa, roasted nuts, roast beef, woody, grass, medical
	2,3‐diethyl‐5methylpyrazine (hazelnut pyrazine)	Roasted nuts, musty, meaty, vegetable, roasted hazelnut
	2‐ethyl‐3,5‐dimethylpyrazine	Roasted nuts
	2‐ethyl‐3,6‐dimethyl‐pyrazine (3,6‐cocoa pyrazine)	Potato, cocoa, roasted, nutty
	2‐methoxy‐3,5‐dimethylpyrazine (3,5‐cocoa pyrazine)	Earthy, burnet, almonds, roasted nuts, coffee
	2‐methoxy‐3,2‐methylpropyrazine	Green, pea green, bell pepper
	2‐methoxy‐3‐isopropylpyrazine	Earthy, pea, bean
	2‐ethyl‐2ethyl‐5‐methylpyrazine	Earthy
	6,7‐dihydro‐5‐methyl‐5H‐cyclopentapyrazine	Roasted nuts, earthy, baked potato, peanut, roasted
	Ethylpyrazine	Peanut butter, musty, nutty, woody, roasted cocoa
Pyridine	Pyridine	Fishy
	Pyrrole	Sweet, nutty, ethereal
	1‐methylpyrrole	Smoky, woody, herbal, negative notes; defective beans
Terpene	Linalool	Flowery, citrus, orange, Terpene, waxy, rose
	Limonene	Citrus, herbal, Terpene, camphor
	Geraniol	Sweet, floral, fruity, rose, waxy, citrus

*Source*: Yeretzian et al. (2019).

Moreover, some volatile compounds formed during coffee roasting are used as a marker to identify the effect of roasting on the coffee. 1H‐indole is used as an indicator of light roast effect, 4‐ethyl‐2‐methoxyphenol as a scorched effect on coffee, phenol as a dark effect of roasting, 3‐hydroxy‐2‐methylpyran‐4‐one (maltol) as a baked effect of roasting, and 2,5‐dimethylfuran as an underdeveloped defect marker of roasting. However, according to different scientific findings, all of the volatile aroma compounds of coffee are not necessary for the aroma of roasted coffee and 12 of them have been identified as critical for the aroma of coffee. Table 3 depicts volatile compounds with their respective aromas and their absence in coffee individually or as a group has a significant effect on the aroma (Yeretzian et al., 2019).

**TABLE 3:** Key compounds responsible for coffee aroma.

Volatile compound	Aroma descriptor
2‐Methylpropanal	Sweet caramel notes
2‐Methylbutanal	Sweet, caramel notes
3‐Methylbutanal	Sweet, caramel notes
2‐Ethyl‐3,5‐dimethylpyrazine	Earthy notes
2‐Ethnyl‐3,5‐dimethylpyrazine	Earthy notes
2,3‐Diethyl‐5‐methylpyrazine	Earthy notes
3‐Isobutayl‐2‐methoxypyrazine	Earthy notes
2‐Furfunylthiol	Roasty, sulfury notes
4‐Vinylguaiacol	Phenolic
Acetaldehyde	Fruity, flowery
Propanal	Fruity flowery
5‐Ethyl‐3‐hydroxy‐4‐methyl‐2(5H)‐furanone/furaneol	Sharp

*Source*: Yeretzian et al. (2019).

## 5 COFFEE AROMA IMPACT COMPOUNDS AND THEIR HEALTH BENEFITS

### 5.1 Caffeine

Within 45 min of ingestion, caffeine is entirely absorbed by the small intestine and stomach and reaches its peak blood levels between 15 and 120 min later. Almost all (99%) of the ingested caffeine is absorbed into the bloodstream (Acikalin & Sanlier, 2021; DePaula & Farah, 2019). It is transported throughout the body after being absorbed and is then digested in the liver. In the liver cells, caffeine is metabolized to produce uracil derivatives, dimethyl and monomethylxanthines, and dimethyl and monomethyluric acids (Arnaud, 2011). The majority of caffeine and other methylxanthine metabolism processes are carried out by phase I (cytochrome P450 CYP) enzymes, particularly CYP1A2, a significant P450 enzyme that makes up about 13% of the total content of this enzyme group in the human liver. Nearly 90% of caffeine metabolism is carried out by the CYP1A2 isoform (Arnaud, 2011; DePaula & Farah, 2019; Djordjevic et al., 2008).

The other routes involve the actions of monooxygenase, *N*‐acetyltransferase, CYP1A1, CYP2E1, and CYP2A6, among others (Burdan, 2015; Lima & Farah, 2018). The predominant caffeine metabolites, methylated xanthines and methyluric acids, are eliminated in urine, whereas paraxanthine is the main caffeine metabolite in plasma. The first step in caffeine CYP1A2 metabolism is 3‐demethylation, which results in 1,7‐dimethylxanthine (paraxanthine), which accounts for approximately 84% of the major caffeine metabolites. Caffeine may potentially be converted to theobromine (12%) by the CYP1A2 enzyme (DePaula & Farah, 2019). CYP2E1 catalyzes the metabolism of theobromine and theophylline (4%) by 7‐and 1‐demethylation of caffeine (Burdan, 2015). Caffeine can be transformed to 1,3,7‐methyluric acid, but to a lesser extent. The main primary caffeine metabolite, paraxanthine, may be demethylated by CYP1A2 to produce the main metabolite, 1‐methylxanthine (70%), which may then be oxidized to 1‐methyluric acid by xanthine oxidase (Lima & Farah, 2018).

Secondary metabolites, such as 7‐methylxanthine (20%), may also be metabolized to 7‐methyluric acid (Arnaud, 2011; Burdan, 2015). Likewise, paraxanthine can be acetylated by *N*‐acetyltransferase 2 to produce 5‐acetylamino‐6‐formylamino‐3‐methyluracil, an unstable molecule that can be nonenzymatically deformylated to produce 5‐acetylamino‐6‐amino‐3‐methyluracil, or it can be hydroxylated by CYP2A6 to produce 1,7‐dimethyl (Acikalin & Sanlier, 2021; DePaula & Farah, 2019; Lima & Farah, 2018).

Caffeine consumption and its direct effects are clearly influenced by genetics. This modulation occurs at both the pharmacodynamic and pharmacokinetic levels, and it has been linked to caffeine response variability (Nehlig, 2018). Caffeine metabolism varies greatly between individuals, owing primarily to differences in CYP1A2 enzyme activity, and there has recently been renewed interest in identifying polymorphisms that influence caffeine metabolism. Some variation in CYP1A2 activity can be attributed to genetic polymorphisms in the CYP1A2 gene, which can result in increased or decreased enzyme inducibility (Grosso & Bracken, 2005). A gene polymorphism explains a large portion of the variability in CYP1A2 activity. After caffeine consumption, an A to C substitution at position 163 (rs762551) in the CYP1A2 gene reduces enzyme inducibility as measured by plasma or urinary caffeine‐to‐metabolite ratios (Nehlig, 2018).

The stimulation of brain activity, mood improvement, and physical performance are the most well‐known short‐term effects of caffeine consumption. Nevertheless, over the past decades, a number of epidemiological studies have found a link between moderate coffee consumption and a lower relative risk of developing chronic degenerative diseases. Moreover, it is reported to have many of these advantages, such as a decreased risk of Alzheimer's and Parkinson's diseases (PDs), and there are also hepatoprotective effects via anti‐inflammatory and antioxidant functions (Gökcen & Şanlier, 2019; Tofalo et al., 2016).

#### 5.1.1 Psychostimulating effect of caffeine

**FIGURE 8:**
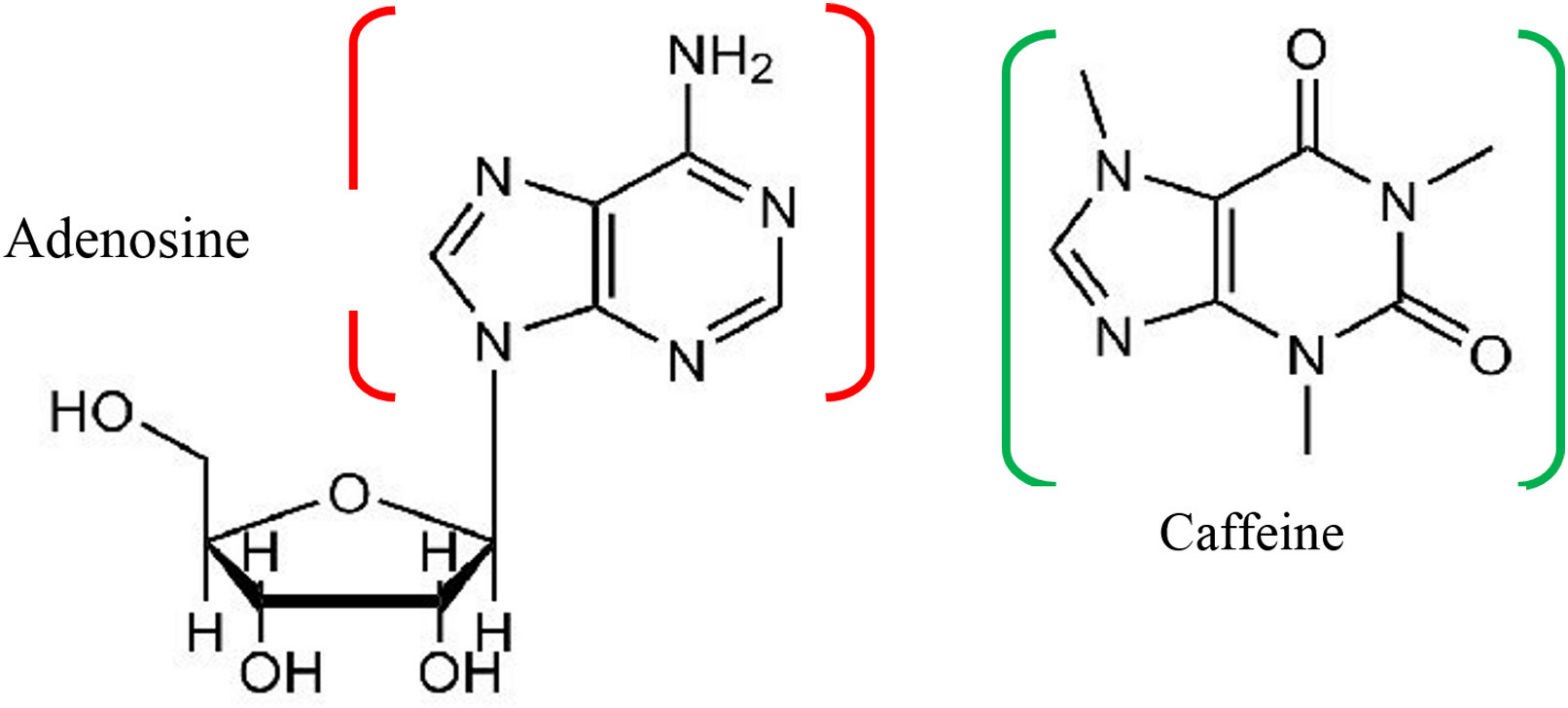
Chemical structures of adenosine receptor and caffeine.

The stomach and intestines can absorb almost all of the caffeine taken and distribute it throughout the body. Once ingested, caffeine will function in a variety of ways in the body, particularly in the brain. It will antagonize adenosine receptors and affect both the peripheral and central nervous systems because of the structural similarities (Figure 8) between adenosine and caffeine (Gaspar & Ramos, 2015). Mainly, it blocks adenosine receptors that allow endogenous adenosine to regulate a wide range of physiological processes (Ferre et al., 2018). Adenosine receptors have several subtypes, including A1, A2A, A2B, and A3 (Effendi et al., 2020). Caffeine A1 subtypes are primarily located in the central nervous system and peripheral nervous system, particularly in the hippocampus, cerebellum, hypothalamus, and cortex. They are also found in the kidneys, lungs, bladder, and heart. Inhibition of the A1 subtype has been proven to reduce anxiety, congestive heart failure, and polarized renal dysfunction. It can help improve cognitive performance and treat Alzheimer's disease (AD) and hypertension (DePaula & Farah, 2019).

**FIGURE 9:**
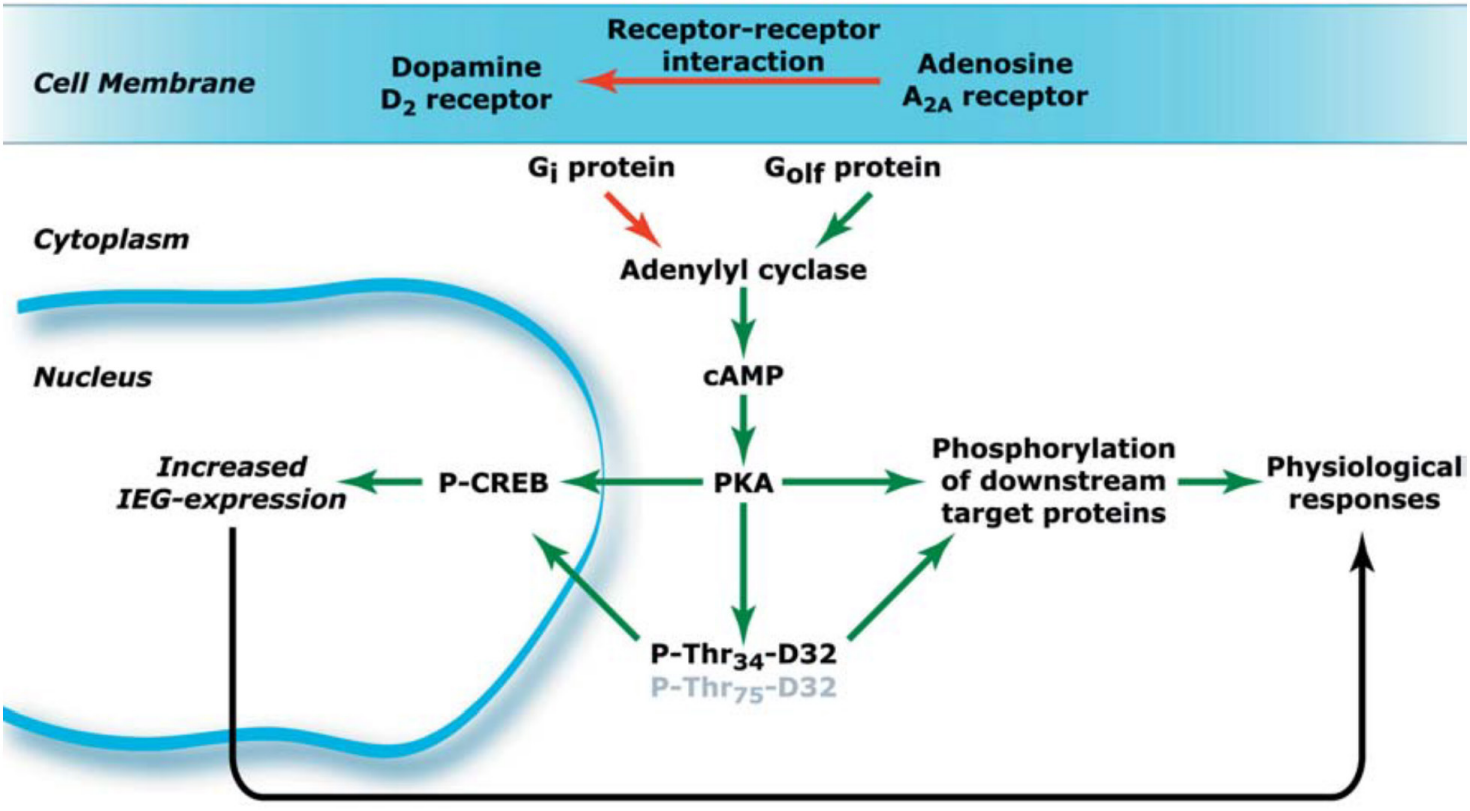
Schematic representation of the antagonistic interactions between adenosine A2A and dopamine D2 receptors in striato‐Gpe projection neurons. At the plasma membrane level, stimulation of A2A receptors results in decreased affinity of the dopamine D2 receptor for agonists. At the cytoplasmic level, A2A receptors stimulate, whereas D2 receptors inhibit the production of cAMP. This results in opposite regulation of the state of phosphorylation of DARPP‐32 and downstream target proteins involved in the control of the activity of striato‐Gpe neurons. In the nucleus, the opposite regulation of the cAMP/PKA pathway results in the opposite regulation of CREB phosphorylation and IEG expression. Green and red arrows indicate positive and negative regulations, respectively (Fisone et al., 2004).

The A2A receptor is mostly found in the dopamine‐rich region of brain tissue, the A2B receptor is primarily found in the digestive system, and the A3 receptor is generally present on the surface of inflammatory cells as well as in the spleen, lungs, heart, and other organs. Numerous clinical and animal studies have demonstrated that caffeine acts as an adenosine receptor inhibitor, altering the expression of various adenosine receptor subtypes and enhancing or attenuating their biological effects. It is most notable for its effects on the A1 and A2A subtypes. Both the A1 and A2A subtypes are linked to caffeine's mental stimulant effects, but the A1 subtype plays a more important role (Effendi et al., 2020; Hu et al., 2019). Caffeine can efficiently suppress the A1 and A2A subtype receptors of microglia in animal models, which lowers the inflammatory environment caused by these cells and ultimately prevents neurodegenerative disorders such as PD (Ren & Chen, 2020). The fundamental framework of new adenosine receptor antagonists is frequently derived from the core structure of caffeine. Acute and chronic caffeine intervention can alter the expression of a number of receptors, including the 5‐hydroxytryptamine (5‐HT) receptor, the cholinergic receptor, the opioid receptor, and the GABA receptor (Hu et al., 2019). The mechanism of action of antagonistic interactions between adenosine A2A and dopamine D2 receptors is shown below in Figure 9.

#### 5.1.2 Anticancer effect of caffeine

Procarcinogens or reactive oxygen species (ROS) damage DNA, which results in the development of cancer. This process involves several steps, such as additional DNA mutations and/or genetic imprints, and is typically accompanied by the activation of protooncogenes, inactivation of tumor suppressor genes, and/or inactivation of genomic stability genes (de Melo Pereira et al., 2020). Caffeine inhibits the release of several procarcinogenic cytokines by increasing the expression of key tumor suppressor proteins, including p16, p21, p53, and Cav‐1 (Al‐Ansari & Aboussekhra, 2014). In another way, caffeine alters the development of cancer via the defense of cells against damage brought on by ROS. In this regard, coffee is viewed as a possible anticarcinogen and may reduce ROS through a direct antioxidant impact (de Melo Pereira et al., 2020).

Moreover, numerous epidemiological studies have shown that caffeine has anticancer properties through reducing the expression of factor‐1, matrix 2‐metalloproteinase, and transforming growth factor in cancer‐associated fibroblasts as well as the level of actin in smooth muscle, which are important markers of myofibroblasts, by inhibiting extracellular signal‐regulated kinases‐1/2 and ‐serine/threonine kinases (Yu et al., 2011).

#### 5.1.3 Caffeine and hepatocellular carcinoma

Consuming coffee is indicated to have a preventative effect on the onset and progression of liver disease. These effects occur in both the presence and absence of the chronic hepatitis B and C virus and are associated with low levels of the liver enzymes such as alanine aminotransferase, aspartate aminotransferase, and gamma‐glutamyl transferase as well as hepatocellular carcinoma (HCC; DePaula & Farah, 2019; Saab et al., 2014). Coffee's hepatoprotective effects are thought to be caused by several processes. In the absence of cellular DNA, oxidized bases accumulate, which is the reason for the link between coffee and HCC. Chromosome instability is a condition caused by oxidative DNA to DNA shortening telomeres (Rudolph et al., 2009). Consuming coffee regularly causes telomere lengthening, which stabilizes chromosomal DNA and stops the evolution of cancer. The presence of caffeine, phenolic metabolites (CGA), as well as other substances such as the diterpenes kahweol and cafestol, all have a role in the mediation of epidemiological research on food (Gressner et al., 2009; Lv et al., 2010). The inverse relationship between coffee consumption and HCC is thought to be due to caffeine's alteration of liver signaling and inflammatory pathways, which lowers circulating levels of inflammatory biomarkers such as interferon‐gamma (IFN‐γ), CX3CL1‐fractalkine, CCL4, FGF‐2, and sTNFRII (de Melo Pereira et al., 2020; Loftfield et al., 2015).

#### 5.1.4 Anti‐inflammatory effect of caffeine

Inflammation is connected with and regulated by a variety of cytokines and chemokines; therefore, lowering these markers should lower the level of total inflammation. Caffeine's ability to reduce inflammation is assumed to be a result of its ability to inhibit phosphodiesterase or to work against adenosine receptors (Lee et al., 2014; Monteiro et al., 2016). Additionally, alterations in the synthesis of cell signaling molecules have been related to caffeine's anti‐inflammatory properties (DePaula & Farah, 2019). Studies portrayed that caffeine enhanced the release of anti‐inflammatory cytokines, such as interleukin 10 (IL‐10; Ouyang et al., 2010; Tauler et al., 2015).

Moreover, caffeine mediates immune suppression of proinflammatory cytokine production, including tumor necrosis factor‐alpha (TNF‐α), interleukin 2 (IL‐2), and interferon‐gamma (IFN‐γ), which are crucial for the onset and progression of autoimmune diseases (DePaula & Farah, 2019). Caffeine has an effect that lowers levels of inflammatory cytokines, which are linked to the immune system. In this respect, a study found that mice's macrophage cells exposed to 0.1–1.2 mM caffeine for 24 h had lower levels of IL‐6, IL‐3, IL‐13, and ROS (Samieirad et al., 2017).

#### 5.1.5 Potential adverse effects of caffeine on mood, behavior, and sleep

Various studies have been conducted on the impact of caffeine on adult mood, and it has been found that the stimulant effects of caffeine are related to the release of neurotransmitters like norepinephrine, dopamine, and serotonin as well as its stimulating effects (Alasmari, 2020). Although ingesting small to moderate amounts of caffeine usually results in pleasant feelings, larger amounts taken all at once or within a short period of time can cause or aggravate jitteriness, insomnia (especially in caffeine abstainers), nervousness, and anxiety (Bertasi et al., 2021; Chaudhary et al., 2016). This is especially true for people who already have psychiatric anxiety disorders, but it can also happen to healthy adults who are not regular caffeine users. Between 400 and 2000 mg of caffeine per day, the range of doses thought to cause anxiety and mood changes differs greatly among writers and government standards (DePaula & Farah, 2019).

In comparison with fast metabolizers, such adverse effects can be observed in slow metabolizers at much lower doses, as low as 50 mg or less (Yang et al., 2010). Nevertheless, after repeated consumption, a tolerance to the general effects of caffeine is frequently seen. Upregulation of adenosine receptors has been suggested as the cause of the poorly understood and highly variable mechanism that increases tolerance in the population (Addicott et al., 2009). Conversely, even in those who are genetically predisposed, tolerance to such an anxiety‐inducing impact develops in adults who use caffeine frequently. A single nucleotide polymorphism in the gene encoding the adenosine A2A receptor has been hypothesized to be the origin of the interindividual heterogeneity in the anxiogenic response to caffeine ingestion (ADORA2A; Erblang et al., 2019). In any case, only a small percentage of caffeine users ingest such large levels, and those who are sensitive to caffeine's effects in general or who experience its anxiogenic effects are likely to abstain from using it (Bertasi et al., 2021; DePaula & Farah, 2019). As a result, caffeine's capacity to cause anxiety in adults is reduced by its self‐limiting nature.

In terms of sleep, single doses of caffeine in adults of roughly 100 mg or more (1.5 mg/kg bw/day in a 70 kg adult) have been shown to shorten sleep duration and increase sleep latency in a dose‐dependent way (DePaula & Farah, 2019; Rodak et al., 2021). This may be followed by a reduction in sleep quality, as seen by a rise in the frequency of unplanned awakenings and movements. Most people appear to not be affected in this way by doses lower than 100 mg. However, people who regularly drink large amounts of caffeine are less likely to report sleep problems than people who only sometimes consume caffeine, which further suggests that people can become tolerant to the effects of caffeine on this parameter (DePaula & Farah, 2019).

### 5.2 Chlorogenic acid

On average, the stomach or upper gastrointestinal tract absorbs one‐third of the CGA consumed by drinking coffee. However, this absorption is partially dependent on hydrolysis, and some of the CGA is broken down by the intestinal microbiota before entering the blood again and being reabsorbed and converted by the liver into ferulic acid, dihydrocaffeic acid, 3‐hydroxyphenylpropionic acid, and coumaric acid (Farah & de Paula Lima, 2019; Nwafor et al., 2022). Additionally, CGA is further conjugated with sulfate, glucuronic acid, or other compounds following hydrolysis or a lack thereof and is absorbed in the colon and other organs (Mateos et al., 2006; Sadeghi et al., 2018). Moreover, in mammals, phase II metabolism or resorption of the metabolites generated by the intestinal flora by the intestine occurs (Clifford et al., 2020; Farah & de Paula Lima, 2019). Finally, the liver absorbs metabolites through blood circulation (Farah & de Paula Lima, 2019; Zeng et al., 2022). CGA is one of the bioactive components of coffee with an antioxidant property that is frequently linked to anti‐inflammatory, antibacterial, antihypertensive, neurodegenerative diseases, and antioxidant activities. In addition, it inhibits the buildup of fat and modifies the metabolism of glucose (Acikalin & Sanlier, 2021; Hu et al., 2019; Zeng et al., 2022).

#### 5.2.1 Antioxidant activity

**FIGURE 10:**
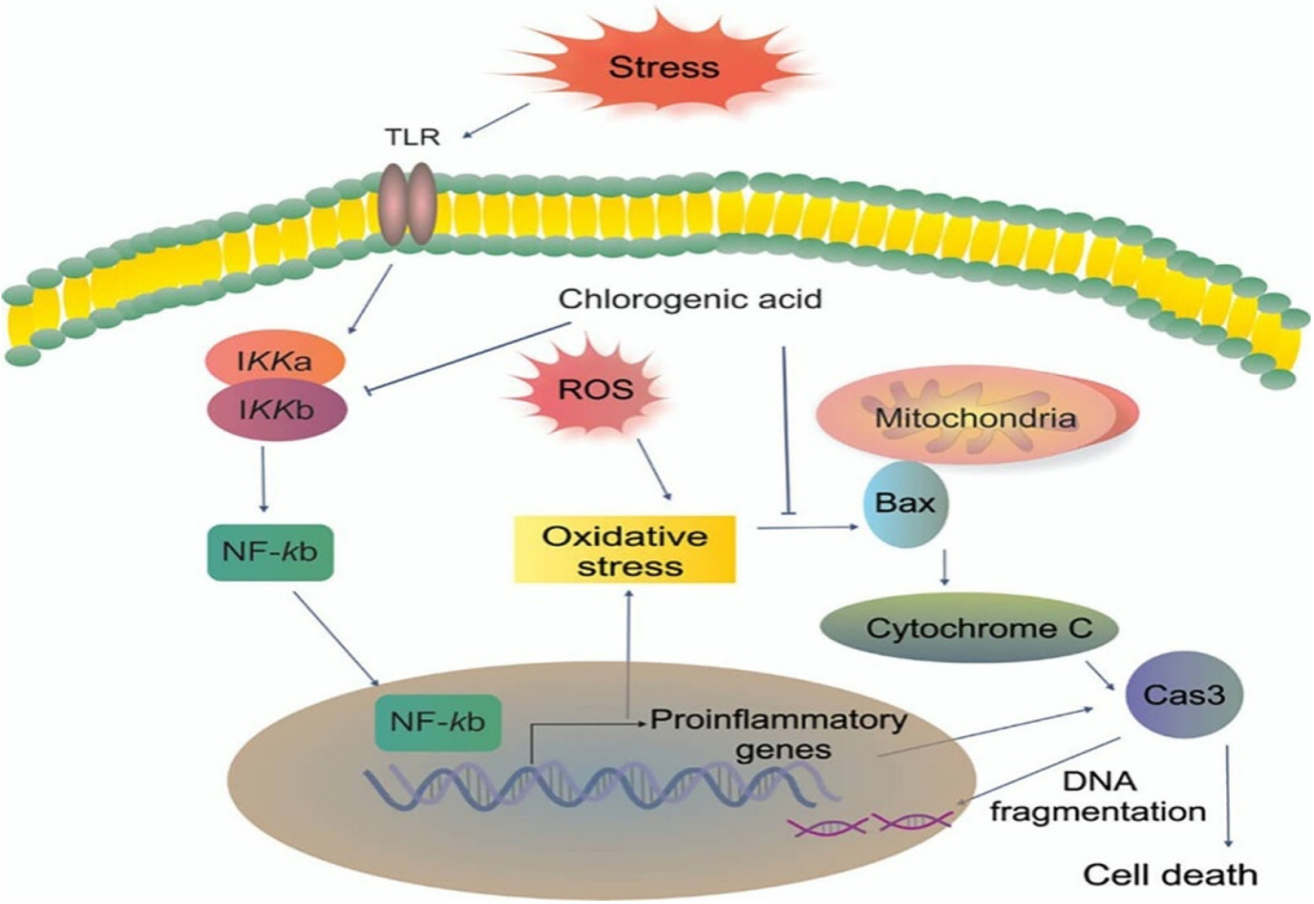
Antioxidant activity mechanism of action of CGA (Rashidi et al., 2022).

Numerous experiments have been done to assess the antioxidant activity of CGAs, both in vitro and in vivo (Cha et al., 2014; Liang & Kitts, 2015; Rojas‐Gonz et al., 2022; Xu et al., 2012). CGAs have a comparable free radical‐scavenging effect as ascorbic acid. Additionally, CGAs have the ability to chelate transition metals like Fe^2+^ to neutralize free radicals and stop chain reactions (Rojas‐Gonz et al., 2022). According to in vitro studies, CGAs may protect against DNA damage as well as the oxidation of low‐density lipoproteins (LDL) brought on by various oxidizing agents (Cinkilic et al., 2013; Gordon & Wishart, 2010). The most significant CGA in coffee, 5‐CQA, has the ability to neutralize peroxynitrite (ONOO‐), superoxide anions (O_2_•‐), hydroxyl radicals (•OH), and 1,1‐diphenyl‐2‐picrylhydrazyl radicals (DPPH; Cha et al., 2014; Rojas‐Gonz et al., 2022), and protect DNA from oxidative stress damage (Liang & Kitts, 2018; Rojas‐Gonz et al., 2022; Xu et al., 2012). Antioxidant activity mechanism of action of CGA is indicated in Figure 10.

#### 5.2.2 CGA and gut health

**FIGURE 11:**
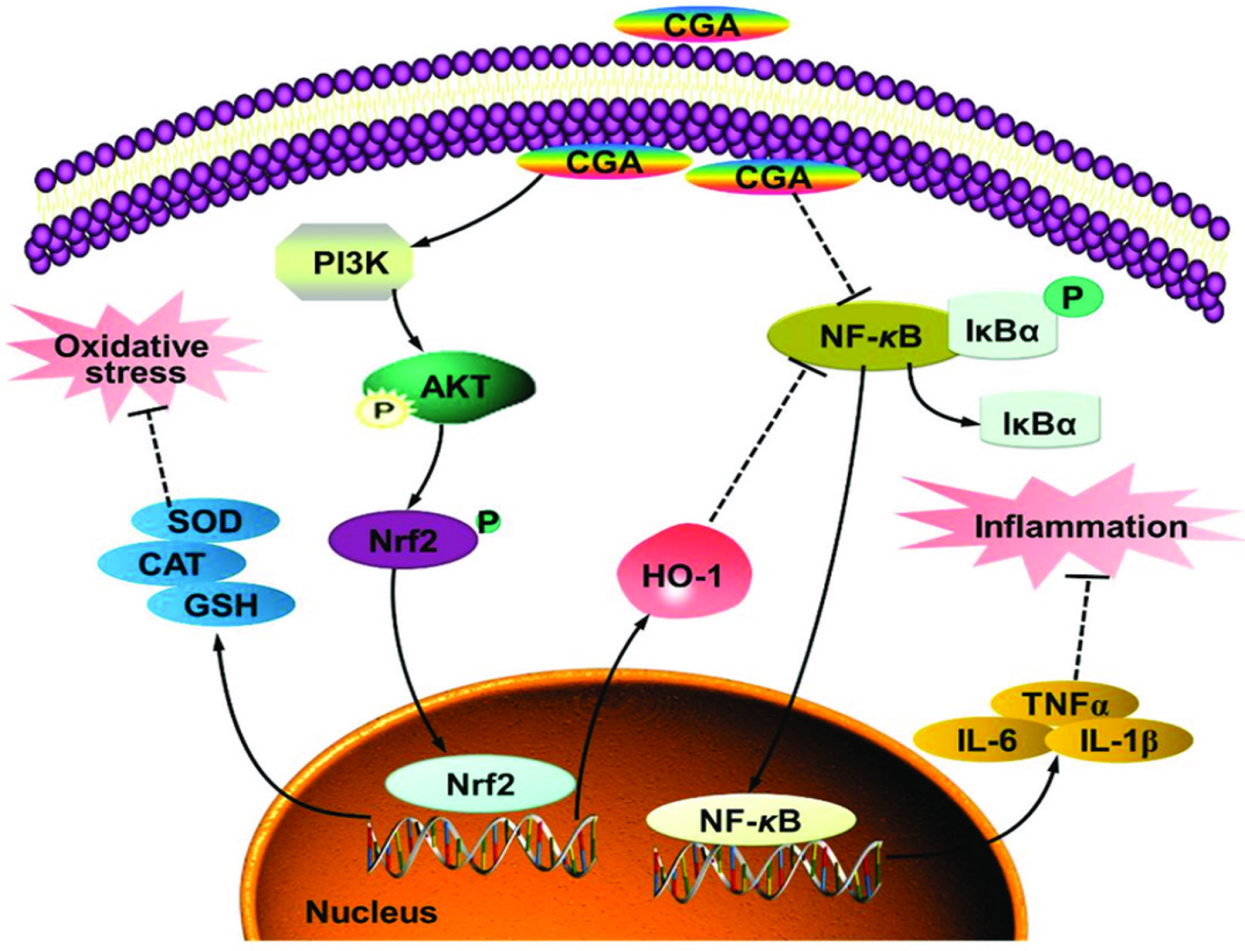
A model of the proposed mechanism of chlorogenic acid (CGA)‐attenuated intestinal inflammation and injury induced by oxidative stress CGA protects the intestinal epithelium against oxidative stress‐induced injury via regulating the phosphatidylinositol‐3‐kinase (PI3K)/protein kinase B (Akt)/nuclear factor erythroid‐derived‐related factor 2 (Nrf2) signaling pathway; CGA attenuates the intestinal inflammation by coregulating the PI3K/Akt/heme oxygenase‐1 (HO‐1) and NF‐kappa‐B inhibitor alpha (Iκ‐Bα)/nuclear factor‐κB (NF‐κB) signaling pathway (Chen et al., 2021).

The intestine is home to tens of thousands of bacteria. They are the primary areas of host–microbiota interactions, cohabit harmoniously with each other's advantages, and support intestine digestion, immunity, and neuronal functions (Zhou et al., 2020). CGA plays a significant “probiotic” role in the gut under the influence of the stomach and gut bacteria, with particular regulatory effects on gut health and the microbiota (Ludwig et al., 2013; Mills et al., 2015). Intestinal inflammation frequently results in impaired intestinal barrier performance and elevated permeability, which encourages the development of illnesses. There are three basic ways that CGA enhances gut barrier performance. First, CGA works by inhibiting mucosal inflammation and the expression of inflammatory factors, including the inhibition of the toll‐like receptor (TLR4)/nuclear factor kappa‐B (NF‐κB) pathway or the phosphorylation of the extracellular regulated protein kinases (ERK1/2)‐signal transducer and activator of antioxidant capacity. It also increases glutathione peroxidase (GSH)‐Px and catalase (CAT; Chen, Xie, et al., 2018; Chen, Yu, et al., 2018; Liang & Kitts, 2018; Qader et al., 2020). Lastly, it encourages intestinal cells to express the tight junction proteins occludin and zonula occludens (ZO)‐1 (Wu et al., 2018; Zeng et al., 2022). Figure 11 depicts proposed mechanism of CGA‐attenuated intestinal inflammation and injury induced by oxidative stress.

#### 5.2.3 Neurodegenerative disease protection

Inflammation and oxidative stress are significant contributors to the incidence and progression of neurodegenerative illnesses like AD and PD (Li et al., 2021). Inflammatory variables cause excessive microglial activation, an overabundance of ROS, glutamate toxicity, and nerve cell injury. CGA reduces oxidative damage brought on by microglia and encourages the inactivation of c‐Src tyrosine kinase in these cells (Socodato et al., 2015). One of the ways CGA controls the abnormal microglia function may be by preventing activation of the NF‐κB/P38 MAPK inflammatory axis and by suppressing the release of inflammatory proteins like TNF‐α and IL‐1β. Additionally, in BV2 microglia, CGA suppresses the expression of nitric oxide synthase (NOS) and COX‐2 in a dose‐dependent way and prevents the production of nitric oxide (Kim et al., 2015).

#### 5.2.4 Role of CGA in type‐2 diabetes and cardiovascular disease

CGA contributes to the metabolism of glucose by promoting glucose uptake in both insulin‐sensitive and noninsulin‐sensitive adiposity (Gökcen & Şanlier, 2019) and also increases insulin secretion, decreases insulin tolerance, and activates or inhibits the body's enzyme activity to promote glucose metabolism, lowering blood glucose levels, and treating diabetes by reducing insulin tolerance (Jin et al., 2015). It is believed that one of the most significant metabolic routes blocks the glucose‐6‐phosphate system, delaying the absorption of glucose in the intestine (Habtemariam, 2019). Additionally, a rat model research revealed that CGAs can directly block blood glucose levels to lower them and by inhibiting the enzyme glucose‐6‐phosphatase involved in hepatic gluconeogenesis (Hu et al., 2019).

It also contributes to the metabolism of lipids by lowering serum and hepatic triglyceride, LDL cholesterol, and LDL oxidation levels, activating lipid metabolism in the liver, and inhibiting lipid absorption in the small intestine (Gökcen & Şanlier, 2019) and encourages lipid metabolism to prevent obesity and heart disease. For example, a significant risk factor in the pathogenesis of arteriosclerosis is the oxidative alteration of LDLs, and an in vivo study with 11 healthy male students indicated that CGAs can protect cardiovascular disorders by reducing the body's level of LDL cholesterol (Yukawa et al., 2004).

**FIGURE 12:**
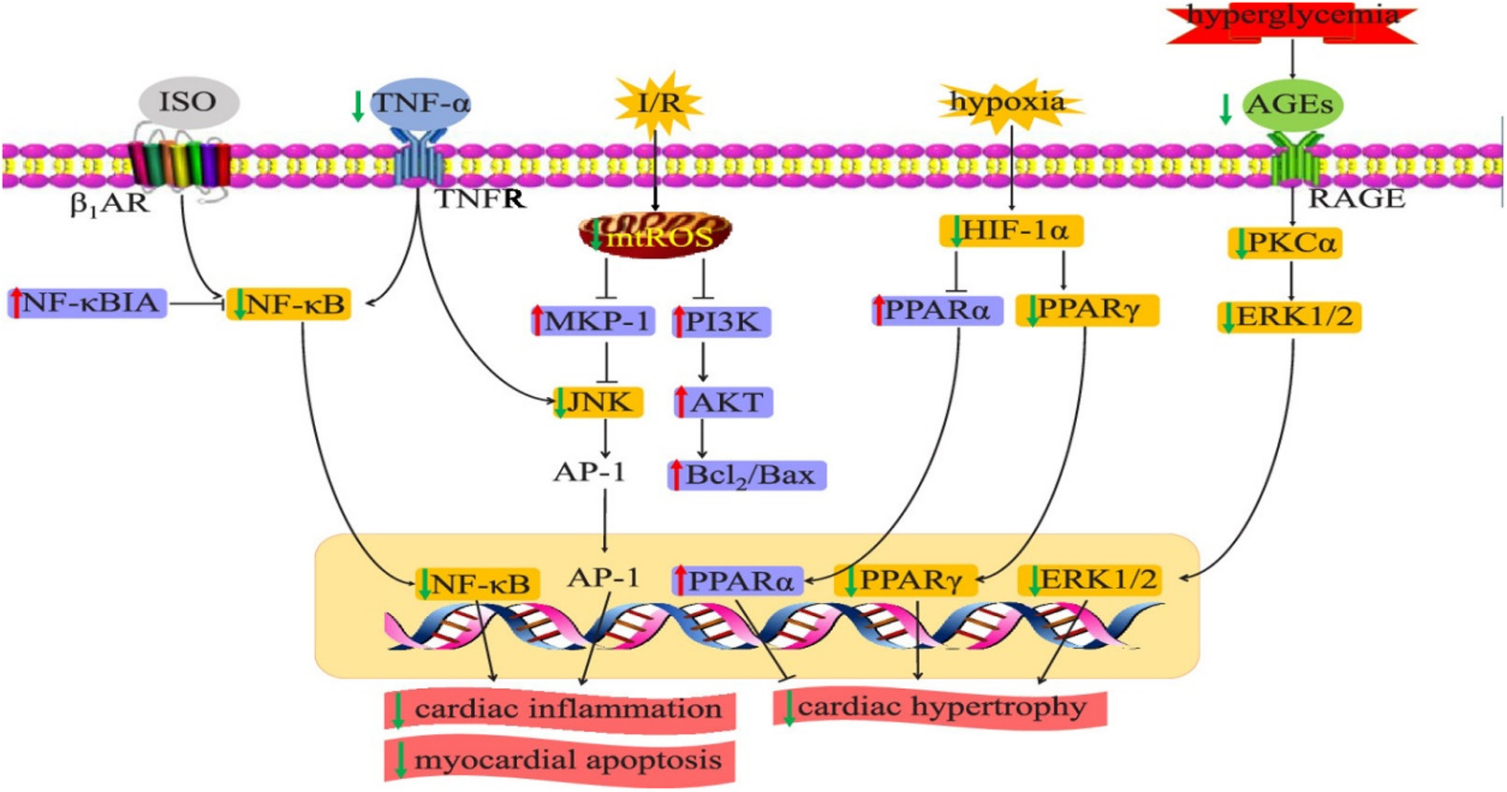
Cardiovascular vascular disease prevention mechanism of action of CGA (Li et al., 2020).

Foam cell production and oxidative stress are the two main causes of the multifactorial inflammatory illness atherosclerosis. By causing cholesterol outflow to lipid‐poor apolipoproteins, such as ApoA1, foam cell development can be prevented. Cholesterol transporters ABCG1 and ABCA1 are crucial in mediating cholesterol efflux to high‐density lipoprotein. Nuclear transcriptional factors LXRα and PPARc control the expression of these molecules (Bobryshev et al., 2016). It has been demonstrated that CGA dramatically raises the mRNA levels of PPAR, LXRα, ABCA1, and ABCG1, as well as PPAR's transcriptional activity. Additionally, a cholesterol efflux assay revealed that the main metabolites caffeic, ferulic, and gallic acids greatly increased cholesterol efflux from RAW264.7 cells. According to these findings, CGA significantly slows the development of atherosclerosis in ApoE/E mice and increases cholesterol efflux from RAW264.7 macrophages (Lukitasari et al., 2021; Wu et al., 2014). CGA cardiovascular vascular disease prevention mechanism of action is indicated in Figure 12.

#### 5.2.5 Anticancer effect of CGA and diterpenes

**FIGURE 13:**
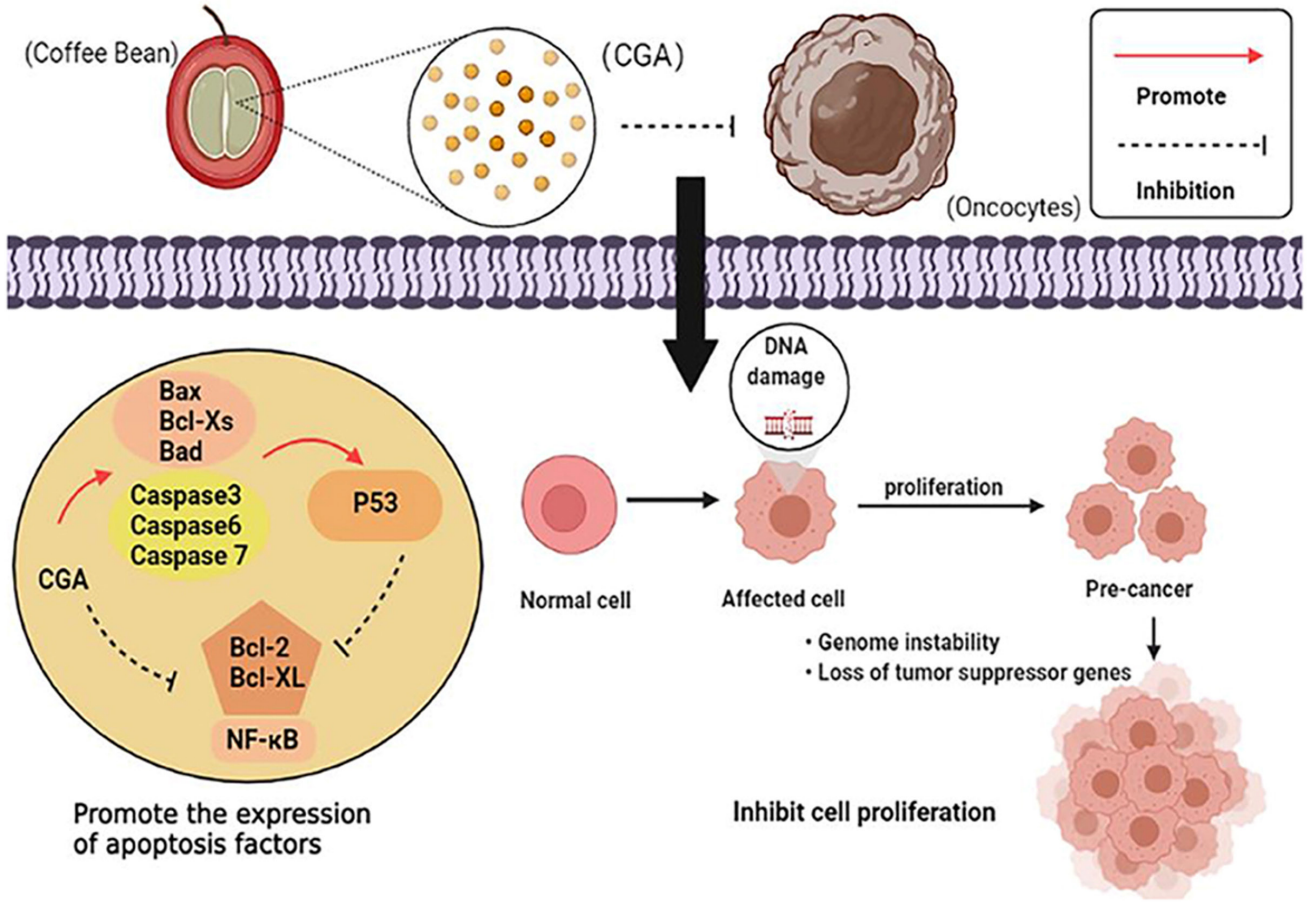
Anticancer effect mechanism of action of CGA (Wang, Pan, et al., 2022; Wang, Wang, et al., 2022).

In combination with diterpenes, CGAs, particularly 5‐CQA, have a significant role in the production of enzymes in phase II metabolism that are responsible for the endogenous antioxidant defenses and anticarcinogenic action by inhibiting DNA methyltransferase (de Melo Pereira et al., 2020). Moreover, CGA, caffeic acid, and kahweol manage inflammatory reactions in the body by preventing the release of specific inflammatory mediators like TNF‐α, IL‐6, and IL‐1 (Nwafor et al., 2022). These proinflammatory biomarkers are activated by the inflammation of tissue induced by signaling activation of NF‐κB. The effectiveness of CGA, caffeic acid, and kahweol is via preventing NF‐κB‐induced activation of proinflammatory biomarkers (de Melo Pereira et al., 2020), As a result, they manage numerous inflammatory diseases, such as acute liver injury, gastrointestinal disease, and endothelial dysfunction (de Melo Pereira et al., 2020; Di Paola et al., 2010; Xu & Chen, 2010). The anticancer effect mechanism of action of CGA is shown below in Figure 13.

### 5.3 Trigonelline

The amount of trigonelline synthesized in the pericarp is significantly higher than that in the seeds, but part of the pericarp‐produced trigonelline can be transferred to the seeds, resulting in a higher level of trigonelline in seeds than in the pericarp. Quinolinic acid, an intermediate formed from the pyrimidine nucleotide, enters the pyridine nucleotide cycle, where it undergoes the following steps: nicotinamide → nicotinic acid → nicotinic acid mononucleotide (NaMN) → nicotinic acid → adenine dinucleotide (NaAD) → NAD → nicotinamide mononucleotide (Zheng et al., 2004).

Trigonelline reported to prevent cardiovascular disease, neuroprotection, and anticarcinogen effects. In diabetic rats, trigonelline controls key enzymes involved in the metabolism of glucose and lipids, including glucokinase, glucose‐6‐phosphatase, fatty acid synthase, and carnitine palmitoyl transferase. This action results in a reduction in blood sugar, cholesterol, and blood lipid levels (Yoshinari et al., 2009; Zhou et al., 2011).

#### 5.3.1 Anticancer effect of trigonelline

Trigonelline has two primary anticancer effects that it achieves without directly killing cancer cells through cytotoxicity. One is to stop cell invasion, which is a key factor in the proliferation of cancer cells (Hirakawa et al., 2005). The other is to control transcription factor Nrf2 expression. In response to cell invasion, the transcription factor Nrf2 is activated to defend cells from damage. Nevertheless, because cancer cells also have the Nrf2 factor, chemotherapy frequently inhibits its activity. Trigonelline can decrease the expression of Nrf2 in cancer cells and enhance the effects of chemotherapy, according to an investigation employed on H6c7 pancreatic duct cells and pancreatic carcinoma cell lines as models (Arlt et al., 2013). In addition, trigonelline has been proven to cross the blood–brain barrier and enter the brain, which can help rats with their memory and have neuroprotective effects but does not fully explain how it works.

#### 5.3.2 Trigonelline in minimizing oxidative stress

Moreover, trigonelline was found to minimize oxidative stress by boosting antioxidant enzyme activity and have a scavenging effect on ROS in a study conducted on rats (Zhou et al., 2013). The activation of the Nrf2 pathway, on the other hand, has been proposed as one of the most crucial defense mechanisms against oxidative stress, and trigonelline defends the organism from oxidative stress by acting as an inhibitor of nuclear factor erythroid 2‐related factor 2 (Nrf2; Boettler et al., 2011). These characteristics explain why trigonelline not only has a favorable impact on immune system cells but also lowers the likelihood of autoimmune disease (Acikalin & Sanlier, 2021).

#### 5.3.3 Trigonelline in inhibition of kidney stone formation

A recent study finding depicts the function of trigonelline in inhibition of kidney stone formation. Trigonelline significantly decreased the size, number, and mass of calcium oxalate monohydrate (COM) crystals during crystallization. The smaller number of COM crystal receptors on the apical membranes of the trigonelline‐treated cells affirmed the downregulation of some of these crystal receptors by trigonelline. Trigonelline also dose dependently inhibited crystal growth and crystal‐cell adhesion. These findings demonstrate how trigonelline inhibits the formation of kidney stones by preventing COM crystallization, crystal growth, and crystal‐cell adhesion via downregulating the crystal receptors on the apical membranes of renal tubular cells. Trigonelline may have a higher inhibitory effect on crystallization than coffee since it has the potential to decrease crystal mass, a more important crystallization index than crystal size (Peerapen et al., 2022).

### 5.4 Diterpenes

Small intestine absorbs about 70% of the amount of cafestol and kahweol consumed (Ren et al., 2019; Socala et al., 2021). Various researches have focused on diterpenes, particularly those describing connections to human health. There have been both positive and negative consequences (Acikalin & Sanlier, 2021). In association with caffeine, the diterpene compounds such as kahweol and cafestol are reported to lower the risk of colon cancer (Lee et al., 2012). However, the diterpenes kahweol and cafestol can have harmful effects on health, such as raising LDL and total cholesterol, and maybe a potential risk of inducing some cardiovascular disease (Godos et al., 2014; Penson et al., 2018; Ren et al., 2019).

**FIGURE 14:**
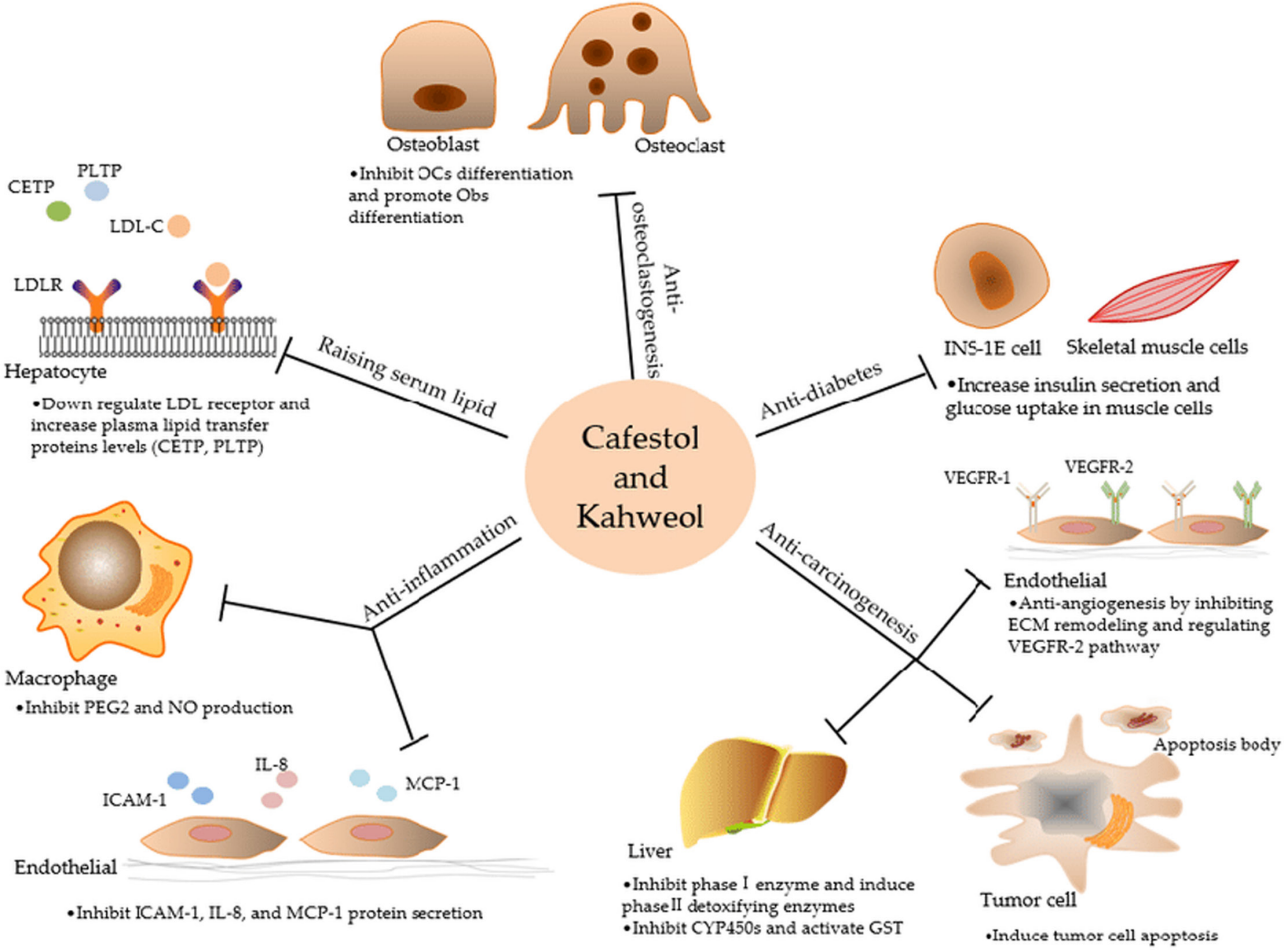
Diagram showing bioactivities and targets of cafestol and kahweol. Cafestol and kahweol raise human serum lipid level and show extensive anti‐inflammatory, anticancer, and potential antidiabetic activities (Ren et al., 2019).

Among the diterpene compounds, cafestol exhibits a cholesterol‐increasing impact by lowering the expression and activity of cholesterol 7‐alpha‐hydroxylase, the rate‐limiting enzyme in the formation of bile acids (Acikalin & Sanlier, 2021; Karabudak et al., 2015). Fortunately, the filtration procedure considerably lowers the amounts of kahweol and cafestol in coffee (Zhang et al., 2012). By removing the foreign elements from the body, filtered coffee can assist the human body defends itself against the damaging effects these compounds have on blood lipids and boost the immune system (Acikalin & Sanlier, 2021). Moreover, numerous researches have shown that cafestol and kahweol have a variety of pharmacological effects, such as anti‐inflammatory, antiangiogenic, and antitumorigenic properties (Acikalin & Sanlier, 2021; Ren et al., 2019; Socala et al., 2021). Figure 14 shows the bioactivities and targets of cafestol and kahweol.

#### 5.4.1 Anticancer effect of diterpenes

The growth and migration of prostate cancer cells were decreased by kahweol acetate and cafestol in a dose‐dependent manner. The treatment combination using kahweol acetate and cafestol synergistically inhibited proliferation and migration of cancer cells in mice. The inhibition is via the induction of apoptosis, prevention of the epithelial–mesenchymal transition, and a decline in androgen receptor. This reduced nuclear androgen receptor in androgen receptor‐positive cells (Iwamoto et al., 2019; Izumi & Mizokami, 2019). Additionally, cafestol and kahweol acetate decreased chemokine receptors (CCR2 and CCR5) without changing their ligands, CCL2 and CCL5. Furthermore, oral treatment of kahweol acetate and cafestol considerably slowed the growth of tumors, according to the results of the xenograft trial (Iwamoto et al., 2019).

#### 5.4.2 Anti‐inflammatory effect of diterpenes

Activated inflammatory corpuscles play a major role in mediating inflammation, which is a protective response of bodies to stimulus. When a pathogen invades, macrophages release proinflammatory cytokines like IL‐1 and TNF‐α or inflammatory mediators such as NO and PGE2, which are essential for enhancing the body's first response. Although this immune response is a protective defense mechanism, excessive inflammation is unmistakably linked to a number of immunological disorders, including cancer, cardiovascular disease, atherosclerosis, autoimmune diseases, allergic reactions, and infectious diseases (El‐Huneidi et al., 2021; Ren et al., 2019).

The activation of various inflammatory signaling pathways by lipopolysaccharide (LPS)‐induced macrophages can be utilized to assess the anti‐inflammatory effects of various medications. LPS‐induced macrophages produce the essential inflammatory modulators NO and PGE2 and the stimulation of iNOS and COX‐2 amplifies their synthesis to a large degree (Rajapakse et al., 2008). In a dose‐dependent manner, cafestol and kahweol can considerably reduce the production of PGE2 and NO in LPS‐activated macrophages. Cafestol and kahweol demonstrated no negative impact on cell viability in cytotoxicity tests performed on RAW 264.7 macrophages (>90% cell viability; Kim et al., 2004; Rajapakse et al., 2008). By decreasing the expression of COX‐2 and iNOS protein as well as the mRNA levels of these enzymes, cafestol and kahweol were found to suppress the production of PGE2 and NO. This was shown by RT‐PCR and WB. They verified that kahweol also showed a sizable anti‐inflammation effect in vivo using a rat model of acute air pouch inflammation brought on by carrageenan (El‐Huneidi et al., 2021; Kim et al., 2006; Ren et al., 2019).

### 5.5 Melanoids

The high MW products of the Maillard reaction often have low bioavailability (Aljahdali & Carbonero, 2019). Melanoidins have been detected in rat urine in both in vitro and in vivo experiments, indicating that melanoidins with lower MW are only partially absorbed (Iriondo‐dehond et al., 2021). However, the majority of melanoidins bypass superior digestion, make it to the colon intact, and are then retrieved in feces (Pastoriza & Rufián‐Henares, 2014; Pérez‐Burillo et al., 2020). Melanoidins can enter the colon intact and serve as substrates for the gut flora, producing chemicals that are connected to antioxidant activity (Alves et al., 2021; Castaldo et al., 2021; Iriondo‐dehond et al., 2021; Pérez‐Burillo et al., 2020; Wang et al., 2011). Before being metabolized by the intestinal microbiota during the digestion of coffee brew melanoidins, polyphenolic compounds can be liberated, leading to the creation of metabolites with improved biological properties relative to the parental molecule (Castaldo et al., 2021).

#### 5.5.1 Antioxidant effect of melanoidins

Melanoidins have been shown to have antioxidant effects on the body in studies using animal models. Their capacity for metal chelation and free radical scavenging may be the mechanism. Additionally, it has been discovered that the copolymerization of phenolic compounds like CGAs accounts for a major portion of their antioxidant effect (Iriondo‐dehond et al., 2021). The maximum antioxidant activity was exhibited by low molecular weight compounds that were liberated from melanoidins following gastrointestinal digestion, even more so than by substances that were ionically linked to melanoidins (Rufian‐Henares & Morales, 2009).

Colon cancer, atherosclerosis, and liver damage are among free radical‐induced disorders that melanoidins effectively protect; this action may be attributed to the bonded CGAs (Daglia et al., 2008; Itzkowitz, 2011). By preventing the release of inflammatory factors, melanoidins can help prevent disorders brought on by bodily inflammation. Additionally, through activating Nrf2‐regulated signaling pathways in a variety of cell types, including intact gut tissue, melanoidins may also boost the antioxidative capability within the cell (Sauer et al., 2013).

Due to their antioxidant properties, melanoidins, both soluble and insoluble, can serve as useful dietary fibers to squelch radical species in the gastrointestinal system. In the upper gastrointestinal tract, in which they can interact with the various microbial species found in the hindgut, melanoidins escape digestion and exhibit the characteristics of soluble dietary fibers. Moreover, a portion of the coffee melanoidins is also fermented by microbes in the gut (Gniechwitz et al., 2008; Reichardt et al., 2009).

#### 5.5.2 Antibacterial effect of melanoidins

Melanoids reported to have an antibacterial effect and three distinct methods were discovered for how they might create antibacterial action through chelation with metal ions: (1) Melanoidins chelate with ions in the medium at low concentrations to mediate antibacterial activity. (2) Melanoidins chelate with the siderophore‐Fe^3+^ complex in bacteria that can produce siderophores, lowering the activity of the bacteria. (3) Higher levels of coffee melanoidin can destroy cell membranes, liberate intracellular molecules, and remove Mg^2+^ from the outer membrane (Rufian‐Henares & de la Cueva, 2009).

Furthermore, melanoidins have been shown to reduce blood pressure by regulating the in vitro activity of the angiotensin‐I‐converting enzyme and to prevent colon cancer by inhibiting the in vitro activity of the family of endo‐peptidases known as matrix metalloproteases (MMPs), which is essential for the development and metastasis of tumors (Rufian‐Henares & Morales, 2009). In living organisms, coffee melanoidins have a variety of biological activities. However, the complexity of their structures significantly restricts our understanding of their biological activities, thus it would be worthwhile to carry out more systematic research studies in the future (Hu et al., 2019).

## 6 DISCUSSION

Various chemical compounds of coffee are reported to have an impact on the perceived aroma of the coffee. Both *C. arabica* and *C. canephora* vary in their biochemical compositions, and they exhibit different flavor profiles. As a result, consumer preferences for coffee types may differ. One of the primary coffee qualities that affect consumers' acceptability is its bitterness. Caffeine is the major aromatic compound of coffee that contributes to its bitterness. The amount of caffeine in roasted Robusta beans doubles that of roasted Arabica beans. This explains a significant percentage of the stronger bitterness often present in coffee brews made with Robusta beans (Seninde & Chambers IV, 2020). Due to this characteristic of the coffee, Arabica coffee has dominated the world coffee market share and is preferred over Robusta. Even though, Arabica and Robusta coffee have been shown to have different levels of biochemical composition, and some studies revealed that their ability to reduce ROS was comparable, but the level of roasting seemed to have the greatest influence on the antioxidant impact (Henrique et al., 2022).

Numerous scientific study reports demonstrated the health‐beneficial effects of coffee chemical compounds, particularly those involved in coffee's perceived aroma (Table 4). They are shown to have a protective effect and reduce the risk of the world's most common public health complications such as type 2 diabetes, cancer, and cardiovascular disease. Moderate intake (3–4 cups/day) of filtered coffee is recommended for a healthy adult to benefit from coffee consumption. However, the amount may vary depending on the physiological condition and health status of the consumer. The caffeine content of one cup of coffee varies depending on geographical region and preparation method. A cup of coffee contains 140 mg of caffeine in Northern Europe and the United Kingdom, 85 mg of caffeine in the United States, and around 50 mg in Southern Europe (Coppi et al., 2023). Consequently, the European Food Safety Authority (EFSA) has established safe levels of caffeine consumption in the general population. Adults should limit their caffeine consumption in one sitting to 200 mg (about two regular 125 mL cups of coffee) and 400 mg throughout the day. Pregnant women should not consume more than 200 mg of caffeine per day, and children and adolescents should not consume more than 3 mg/kg body weight for a 70 kg adult pose no safety concerns. Pregnant women and others who need to limit caffeine consumption can safely drink four cups of some coffees per day without exceeding the recommended caffeine intake; however, drinking even one cup of espresso at the higher end of the scale will exceed the recommended limit of 200 mg/day (Coppi et al., 2023; Nehlig, 2018).

**TABLE 4:** Overview of in vivo and in vitro study on the health benefits of coffee and their proposed mechanism of action.

Coffee bioactive compound	Study	Study type	Proposed mechanism of action	References
Caffeine	Caffeine inhibits cell proliferation and regulates PKA/GSK3β pathways in U87MG human glioma cells	Both in vitro and in vivo	Caffeine (1 mg/mL) treatment inhibited glioma cell proliferation via G0/G1 cell cycle arrest by inhibiting Rb phosphorylation. Caffeine also caused apoptosis by activating caspase‐3 and cleaving poly(ADP‐ribose) polymerase (PARP). Caffeine also phosphorylated glycogen synthase kinase 3 beta (GSK3β) serine 9. Caffeine‐induced GSK3βser9 phosphorylation was inhibited by H89, a pharmacological inhibitor of protein kinase A (PKA), suggesting that the mechanism may involve a cAMP‐dependent PKA‐dependent pathway. Caffeine‐treated tumors had lower proliferation and higher apoptosis in vivo than vehicle‐treated tumors	Ku et al. (2011)
Caffeine	Low concentration of caffeine inhibits the progression of hepatocellular carcinoma	In vitro	Caffeine (<412 μM) may slow the progression of HCC via the Akt signaling pathway	Dong et al. (2015)
Caffeine	Caffeine suppresses the progression of human glioblastoma	In vitro	Caffeine (0.1 and 0.5 mM) inhibited cell invasion in U‐87MG, GBM8401, and LN229 cultures. Caffeine reduced cathepsin B mRNA, protein expression, and activity. Caffeine treatment also increased mRNA and protein expression of tissue inhibitor of metalloproteinase‐1 (TIMP‐1) while decreasing mRNA and protein expression of matrix metalloproteinase‐2 (MMP‐2). Caffeine reduced the expression of Ki67, p‐p38, phosphorylated extracellular regulated protein kinases (p‐ERK), and membranous integrins β1 and β3. The Rho‐associated protein kinase (ROCK) inhibitor Y27632 inhibited caffeine‐mediated reductions in cathepsin B, phosphorylated focal adhesion kinase (p‐FAK), and p‐ERK, as well as invasion. Furthermore, caffeine reduced tumor size, cathepsin B, and Ki67 expression in an animal model. Caffeine inhibited glioma cell invasion and tumor growth in an orthotopic xenograft animal model, indicating that it has anticancer potential in glioma therapy	Cheng et al. (2016)
Caffeine	Caffeine prevents LPS‐induced inflammatory responses in RAW264.7 cells and zebrafish	Both in vitro and in vivo	Caffeine (1000 and 1200 μM) reduced the inflammatory mediator nitric oxide (NO) induced by LPS. Caffeine treatment also reduced the expression of proinflammatory genes such as inducible nitric oxide synthase (iNOS), cyclooxygenase‐2 (COX‐2), interleukin (IL)‐3, IL‐6, and IL‐12, as well as IL‐6 secretion and phosphorylated p38MAPK expression in RAW264.7 cells treated with LPS. Caffeine inhibited nuclear factor κB (NF‐κB) translocation via κ‐IBα phosphorylation. Caffeine also inhibited LPS‐induced NO production in zebrafish	Hwang et al. (2016)
Caffeine	Caffeine shows an anti‐inflammatory activity in Swiss mice	In vitro	The key proinflammatory cytokines IL‐6 and 1L‐1β were reduced by caffeine treatment (0.5 and 5 mg/kg). As a result of the low mRNA expression of iNOS in splenocytes, the involvement of nitric oxide in the bactericidal action was ruled out	Almeida et al. (2021)
Caffeine	Caffeine shows anti‐inflammatory effect on muscle under lipopolysaccharide‐induced inflammation	In vivo	Caffeine (6 mg/kg) pretreatment decreased the expression of Il1b, Il6, and Tnfa while increasing the expression of Il10 and Il13. Caffeine's negative modulation of the inflammatory response included a decrease in inflammasome components, Asc and Casp1, promoting an anti‐inflammatory scenario. Caffeine treatment promoted the upregulation of adenosinergic receptors, Adora1 and Adora2A, which was offset by LPS. Furthermore, there was a significant increase in Adora2A promoter hypermethylation, which could be a compensatory response to increased Adora2A expression	Eichwald et al. (2023)
Chlorogenic acid	Chlorogenic acid shows an anticancer effect	In vitro	CGA (50 μM) significantly reduced HIF‐1β and SPHK‐1 expression as well as SPHK‐1 activity in hypoxia‐induced DU145 cells. Furthermore, CGA inhibited the phosphorylation of AKT and GSK‐3, which are associated with HIF‐1β stabilization, and had a concentration‐dependent effect on SPHK‐1. SPHK‐1 signaling pathway inhibition of HIF‐1β by CGA using SPHK‐1 siRNA and SPHK inhibitor (SKI). CGA inhibited hypoxia‐induced angiogenesis by decreasing VEGF secretion and cellular expression	Lee et al. (2017)
Chlorogenic acid	Chlorogenic acid induces reactive oxygen species generation and inhibits the viability of human colon cancer cells	In vitro	CGA (1000 μmol/L) inhibited HCT116 and HT29 cell viability in a dose‐dependent manner. ROS production was induced by CGA. Furthermore, CGA induced S phase cell cycle arrest and suppressed extracellular signal‐related kinase activation in both cell types, which likely contributes to the ROS‐induced viability inhibition caused by CGA treatment	Hou et al. (2017)
Chlorogenic acid	Chlorogenic acid improves intestinal development in weaned pigs	In vivo	CGA (1000 mg/kg) supplementation reduced serum tumor necrosis factor‐α, interleukin‐6, and interleukin‐1IL‐6 concentrations while increasing serum immunoglobulin G and jejunal secretory immunoglobulin A concentrations in comparison to the control group. CGA, on the other hand, increased jejunal villus height, duodenal and jejunal villus width, and jejunal and ileal villus height/crypt depth. CGA not only reduced the number of duodenal and jejunal cells in the G0G1 phase, but also increased the number of jejunal and ileal cells in the S phase. CGA supplementation reduced the percentages of late and total apoptotic cells in the jejunum, as well as the ratio of B‐cell lymphoma‐2‐associated X protein to B‐cell lymphoma‐2 (Bcl‐2) in the duodenum and jejunum. Finally, CGA increased Bcl‐2 expression in the duodenum and jejunum while decreasing caspase‐3, caspase‐9, and Fas expression in the jejunum and ileum	Chen, Xie, et al. (2018, Chen, Yu, et al., 2018)
Chlorogenic acid	Chlorogenic acid inhibits growth of 4 T1 breast cancer cells	In vitro	Chlorogenic acid (200 μM) increased Bax expression while decreasing Bcl2 expression in 4 T1 cells. P53 and caspase3 expression increased in 4 T1 cells	Changizi et al. (2020)
Chlorogenic acid	Chlorogenic acid shows antitumor activity	In vitro and in vivo	CGA (80 μM) inhibits the phosphorylation of p‐protein kinase C alpha (PKCα), Akt, and the expression of Rictor and F‐Actin, all of which are known to activate cell growth and organize the Actin cytoskeleton	Tan et al. (2019)
Chlorogenic acid	Chlorogenic acid inhibits the proliferation of human lung cancer A549 cell lines	Both in vitro and in vivo	Chlorogenic acid (400 μM) inhibits the binding of annexin A2 to the p50 subunit, thereby inhibiting the expression of downstream antiapoptotic genes cIAP1 and cIAP2 of the NF‐B signaling pathway in A549 cells. Furthermore, chlorogenic acid inhibited the binding of annexin A2 to Actin, potentially inhibiting tumor cell cycle and migration	Wang et al. (2020)
Chlorogenic acid	Chlorogenic acid inhibits proliferation in human hepatoma cells	In vitro	CGA (300 μM) effectively suppressed the noncanonical NF‐B signaling pathway while activating HCC mitochondrial apoptosis via upregulation of the BH3‐only protein Bcl‐2 binding component 3 (BBC3)	Jiang et al. (2021)
Trigonelline	Trigonelline protects the cardiocyte from hydrogen peroxide‐induced apoptosis in H9c2 cells	In vitro	The addition of TG (25–100 μM) to cells reduced H_2_O_2_‐induced cell death and increased antioxidant activity. Furthermore, during H_2_O_2_‐induced oxidative stress, TG regulated the apoptotic genes caspase‐3 and caspase‐9, as well as the antiapoptotic genes Bcl‐2 and Bcl‐XL	Ilavenil et al. (2015)
Trigonelline	Trigonelline reduced diabetic nephropathy and insulin resistance in type 2 diabetic rats	In vivo	In T2DM rats, trigonelline (40 mg/kg/day) induced the protein expression of peroxisome proliferator‐activated receptor (PPAR) and suppressed the protein expression of glucose transporter 4, but suppressed the protein expression of tumor necrosis factor and leptin	Li et al. (2019)
Trigonelline	Trigonelline‐loaded micelles on Nrf2 suppression to overcome oxaliplatin resistance in colon cancer cells	In vitro	Trigonelline micelles (500 nM) reduced Nrf2 mRNA expression in resistant colon cancer cells by nearly twofold. Furthermore, trigonelline reduced Ho‐1 mRNA expression threefold	Pirpour Tazehkand et al. (2020)
Trigonelline	Trigonelline induces autophagy to protect mesangial cells in response to high glucose	In vitro	In HMCs induced by high glucose, TG (800 μM) improved proliferation, increased the expression of miR‐5189‐5p, decreased HIF1AN, and restored autophagy inhibition. HIF1AN expression was inhibited by miR‐5189‐5p mimics, while HIF1AN expression was increased by miR‐5189‐5p inhibitor. MiR‐5189‐5p mimics significantly improved HMC proliferation induced by high glucose, reduced p‐AMPK, SIRT1, LC3B, and Beclin‐1 relative protein expression, and significantly increased p62 relative protein expression	Chen, Luo, et al. (2021), Chen, Ma, et al. (2021)
Trigonelline	Trigonelline inhibits Nrf2 via EGFR signaling pathway and augments efficacy of Cisplatin and Etoposide in NSCLC cells	In vitro	In both A549 and NCIH460 cells, trigonelline (50 μM) reduced nuclear accumulation of pNrf2 by fourfold and downregulated Nrf2 targeted genes. Trigonelline inhibited Nrf2 by reducing EGFR signaling pathway activation and its downstream effector ERK 1/2 kinase. Trigonelline in combination with Cisplatin/Etoposide inhibited NSCLC cell proliferation (A549, NCIH460, and NCIH1299) without causing cytotoxicity in normal lung epithelial cells (L132)	Fouzder et al. (2021)
Trigonelline	Trigonelline prevents kidney stone formation processes	In vitro	Trigonelline (100 μM)) significantly reduced COM crystal size, number, and mass during crystallization by inhibiting calcium oxalate crystallization, growth, and crystal‐cell adhesion and downregulating crystal receptors. Furthermore, trigonelline inhibited crystal growth and crystal‐cell adhesion in a dose‐dependent manner	Peerapen et al. (2022)
Trigonelline	Trigonelline prevents ultraviolet‐B‐induced oxidative DNA damage in primary human dermal fibroblasts and BALB/c mice	Both in vitro and in vivo	TG (50 μM) protects HDF cells and BALB/c mice from UV‐B‐induced DNA damage by regulating the expression of key DNA damage protein markers such as P53, ATM, ATR, H2AX, Chk1, and Chk2. In immunocytochemistry, TG provides geno‐protection to UV‐B‐irradiated HDFs by alleviating CPD induction, reducing the number of TUNEL‐positive cells, and decreasing the expression levels of the DNA damage marker protein H2AX. TG also protects against UVB‐induced oxidative stress by activating the PI3K‐AKT‐Nrf2 signaling pathway. TG protects the genome from UV‐B irradiation via the PI3K‐AKT‐Nrf2 signaling pathway	Tanveer et al. (2023)
Diterpene kahweol	Coffee diterpene kahweol protect human dopaminergic neurons from 6 hydroxydopamine‐derived oxidative stress	In vitro	Kahweol (10 μM) significantly reduced 6‐OHDA‐induced ROS production, caspase‐3 activation, and cell death. Kahweol also increased heme oxygenase‐1 (HO‐1) expression, which protected neurons from 6‐OHDA‐induced oxidative injury. In addition, kahweol activated PI3K and p38, which are involved in the induction of Nrf2, HO‐1 expression, and neuroprotection. These findings suggest that the PI3K and p38/Nrf2 signaling pathways control the intracellular levels of ROS by regulating the antioxidant enzyme HO‐1	Hwang and Jeong (2008)
Cafestol and kahweol	Coffee, cafestol, and kahweol induce apoptosis through regulation of specificity protein 1 expression in human malignant pleural mesothelioma	In vitro	In MSTO‐211H cells, cafestol (60 and 90 μM) and kahweol (40 and 60 μM) increased sub‐G1 population and nuclear condensation. Sp1 inhibitor Mithramycin A previously confirmed Sp1 roles in cell proliferation and apoptosis of MSTO‐211H cells. Sp1 protein levels were significantly suppressed by cafestol and kahweol. In MSTO‐211H cells, kahweol slightly reduced Sp1 mRNA, whereas cafestol had no effect. In mesothelioma cells, cafestol and kahweol modulated the promoter activity and protein expression levels of Sp1 regulatory genes such as cyclin D1, Mcl‐1, and survivin. Cafestol‐induced cleavages of Bid, caspase‐3, and PARP, as well as kahweol‐induced upregulation of Bax and downregulation of Bcl‐xl, activated the apoptosis signaling cascade	Lee et al. (2012)
Diterpene kahweol	Coffee diterpene kahweol suppresses cell proliferation in human colorectal cancer cells	In vitro	Kahweol (50 μM) reduced the protein level of cyclin D1 in HCT116 and SW480 cells. MG132 treatment reduced kahweol‐mediated cyclin D1 downregulation, and cyclin D1 half‐life was reduced in kahweol‐treated cells. Kahweol increased cyclin D1 phosphorylation at threonine‐286, and a point mutation of threonine‐286 to alanine reduced kahweol‐induced cyclin D1 degradation. PD98059, SP600125, or LiCl inhibition of ERK1/2, JNK, or GSK3b inhibited cyclin D1 phosphorylation and downregulation by kahweol	Park et al. (2016)
Diterpenes kahweol acetate and cafestol	Coffee diterpenes kahweol acetate and cafestol synergistically inhibit the proliferation and migration of prostate cancer cells	In vivo	Cafestol (30 μM) and kahweol (30 μM) acetate inhibited proliferation and migration by inducing apoptosis, inhibiting epithelial–mesenchymal transition, and decreasing androgen receptor, resulting in a decrease in nuclear androgen receptor in androgen receptor‐positive cells. Furthermore, kahweol acetate and cafestol inhibited CCR2 and CCR5 while increasing their ligands, CCL2 and CCL5. The xenograft study found that taking kahweol acetate and cafestol orally significantly reduced tumor growth	Iwamoto et al. (2019)
Diterpene, Kahweol	Coffee Diterpene, Kahweol, Ameliorates Pancreatic β‐Cell Function	In vitro	Kahweol (5 μM) downregulated STZ‐induced nuclear factor kappa B (NF‐κB), and the antioxidant proteins, heme oxygenase‐1 (HMOX‐1), and inhibitor of DNA binding and cell differentiation (Id) proteins (ID1, ID3) while upregulated protein expression of insulin (INS), p‐AKT, and B‐cell lymphoma 2 (BCL‐2)	El‐Huneidi et al. (2021)
Melanoids	Melanoidins from coffee, scavenge α‐dicarbonyl compounds under simulated physiological conditions	In vitro	Caffeic acid (CA) and 3‐caffeoylquinic acid (1 mg/mL) had DC‐scavenging kinetic profiles similar to HMW‐CM, and mass spectrometry data confirmed that hydroxyalkylation and aromatic substitution reactions resulted in the formation of a stable adduct between CA and MGO	Zhang et al. (2019)

Quinic acid is a type of phenolic acid found in a variety of plants and microorganisms. It is impossible for mammals, including humans, to synthesize (Dong et al., 2022). The natural isomer is 1 L‐(−)‐quinic acid; despite this, it is ambiguously named 1D‐quinic acid in some scientific literature (Abrankó & Clifford, 2017). Consumption of acyl‐quinic acid sources, such as coffee, has been linked to improved cardiovascular and metabolic health (Domínguez‐Fernández et al., 2022). 5‐O‐CQA, a known dietary precursor of this metabolite (3′‐methoxycinnamic acid‐4′‐sulfate), has been shown in clinical studies to reduce diastolic and systolic blood pressure, and beverages containing acyl‐quinic acids have produced an acute improvement in flow‐mediated dilatation, implying that many people who drink such beverages regularly may gain at least a modest health benefit long term (Rubió et al., 2021). Furthermore, epidemiological and intervention research suggests that they may lower the risk of developing type 2 diabetes and cardiovascular disease (Clifford et al., 2020). Additionally, 1 L‐(−)‐quinic acid has been shown to have antioxidant and anti‐inflammatory properties. It stimulates the production of tryptophan and nicotinamide in the intestine, which raises the concentrations of all lipoproteins (Dong et al., 2022).

Despite the positive health impacts of consuming coffee, too much/excessive intake from the recommended daily intake is directly associated with potential health risks. It may increase the possibility of cardiovascular disease, lung cancer, interaction of some coffee bioactive components with drugs and nutrients, and adverse pregnancy‐related complications. Furthermore, intake of unfiltered coffee may also be prone to a considerable amount of diterpenes such as cafestol and kahweol. They are known to raise the blood LDL, the total cholesterol level, and the potential risk for cardiovascular disease. In another way, some epidemiological study findings on the health benefits of coffee consumption have limitations, particularly the doses used in animal and in vitro studies to benefit from coffee consumption, which are not easily achievable in normal human consumption.

Epidemiological studies conducted in the 1980s and 1990s linked coffee consumption with poor health, heart disease, cancer, infertility, and other issues. The degree of association between coffee consumption and the occurrence of each of these diseases and conditions has been low and inconsistent. The inconsistencies in the relationship between coffee or caffeine consumption and the occurrence of various diseases may be due to methodological differences between studies. The precision of the various instruments used to assess coffee and caffeine intake could be a significant difference. Coffee intake estimation only requires precise measurement of the volume of coffee consumed in a given time period, whereas caffeine intake measurements are much more complex because they include not only the volume of coffee consumed, but also the caffeine concentration of the coffee, and the consumption of caffeine from sources other than coffee.

Another possible explanation for discrepancies in study findings is the presence of certain disease risk factors that are linked to both coffee or caffeine consumption and the disease under investigation. In addition, there was no control for the effects of confounding variables, and most studies simply asked study participants to report how many cups of caffeinated coffee they drank on a daily basis. Any dose–response relationships that may have existed could have been obscured by the imprecision of such estimates. Furthermore, coffee consumption epidemiological studies often fail to include information on the genetic type and source of coffee beans, the type and brand of coffee, the blend and mode of preparation, the caffeine or another physiologically active or toxic component concentration, and the volume of coffee per serving.

## 7 CONCLUSIONS

Bioactive compounds in coffee and their role in lowering the risk of major public health problems, as well as their mechanisms of action, are addressed in this article. Coffee Arabica and Robusta are the two most widely consumed coffee species for their pleasant aroma and flavor. They contain different mixtures of aroma‐impact compounds, such as caffeine, CGA, trigonelline, melanoidins, and diterpenes that significantly determine the perceived quality of coffee. Study findings show that these bioactive compounds are associated with a low risk of type‐2 diabetes, cardiovascular disease, neurodegenerative diseases, and various types of cancer. Moreover, they were reported to enhance lipid and glucose metabolism, block the production of inflammatory mediators, and avoid damage from free radicals. Based on an individual's physiological condition and health status, three to four cups of filtered coffee per day have been associated with cardiovascular protection and other health benefits. However, the amount of caffeine and other bioactive compounds in a cup of coffee varies depending on the geographical origin and method of preparation. As a result, the EFSA recommends 400 mg of caffeine per day for adults. Caffeine consumption should be limited to no more than 200 mg per day for pregnant women. However, even one cup of espresso at the higher end of the scale exceeds the recommended daily limit of 200 mg. In general, exceeding the recommended daily allowance, calcium and vitamin D deficiency, and drinking unfiltered coffee all increase the risk of potential health risks, including cardiovascular disease. Moderate coffee consumption is advised to reduce the risk of a variety of noncommunicable diseases.

## AUTHOR CONTRIBUTIONS

**Markos Urugo Makiso:** Conceptualization (equal); methodology (equal); writing – original draft (equal). **Yetenayet Bekele Tola:** Investigation (equal); supervision (equal); writing – review and editing (equal). **Onwuchekwa Ogah:** Conceptualization (equal); investigation (equal); methodology (equal); supervision (equal); writing – review and editing (equal). **Fitsum Liben Endale:** Data curation (equal); investigation (equal); validation (equal); writing – review and editing (equal).

## CONFLICT OF INTEREST STATEMENT

The authors declare that they do not have any conflict of interest.

## ETHICS STATEMENT

This study does not involve any human or animal testing.

## Data Availability

The data that support the findings of this study are available from the corresponding author upon reasonable request.
